# Machine learning-powered discovery of a novel berberine derivative inducing SCD-dependent ferroptosis in osteosarcoma

**DOI:** 10.1186/s12967-025-07358-6

**Published:** 2025-11-20

**Authors:** Mingyu He, Yanyan Liu, Tao Li, Ying Liu, Xinyue Wang, Jiajie Xie, Ao Wang, Yanquan Wang, Ye Yuan, Min Cui, Zhimin Du

**Affiliations:** 1 Guangdong Provincial Key Laboratory of Tumor Interventional Diagnosis and Treatment, Zhuhai People’s Hospital (The Affiliated Hospital of Beijing Institute of Technology, Zhuhai Clinical Medical College of Jinan University), Zhuhai, Guangdong 519000 China; 2 Departments of Pharmacology (The State-Province Key Laboratories of Biomedicine Pharmaceutics of China, Key Laboratory of Cardiovascular Research Ministry of Education), State Key Laboratory of Frigid Zone Cardiovascular Diseases (SKLFZCD), College of Pharmacy, Harbin Medical University, Harbin, 150081 China; 3 Department of Pharmacy at the Second Affliated Hospital, State Key Laboratory of Frigid Zone Cardiovascular Diseases (SKLFZCD), Harbin Medical University, Harbin, 150081 China; 4 Academician Collaborative Laboratory for Basic Research and Translation of Chronic Diseases, Central Laboratories, the First Affiliated Hospital of Jinan University, Jinan University, Guangzhou, 511436, China; 5 State Key Laboratory of Mechanism and Quality of Chinese Medicine, Macau University of Science and Technology, Macau, 999078 China

**Keywords:** Berberine derivative, 9-O-methoxyethylberberrubine bromide, Osteosarcoma, Machine learning, SCD, NEDD4L, Ferroptosis

## Abstract

**Background:**

Despite decades of therapeutic development, osteosarcoma survival remains poor. Although berberine (BBR) shows anti-tumor activity, its efficacy is limited. We addressed this through structural modification and machine learning-guided discovery, developing a novel derivative: 9-O-methoxyethylberberrubine bromide (B1).

**Methods:**

In vivo, subcutaneous and orthotopic models were established in BALB/c nude mice using 143B cells. Treatment groups received daily B1 (0.1-5 mg/kg) or berberine (5, 50 mg/kg); a positive control group received doxorubicin (1 mg/kg). Tumor growth was assessed by volume and weight; tissue necrosis, proliferation, and apoptosis were analyzed. In vitro, human osteosarcoma cells (143B, U2OS, HOS) and human bone marrow mesenchymal stem cells (hBMSCs) were treated with B1, and anti-proliferation was evaluated via CCK-8, EdU, colony formation, and transwell assays. We integrated machine learning into our proteomic discovery pipeline to prioritize critical targets. Proteomic sequencing was followed by multi-algorithm feature selection including least absolute shrinkage and selection operator (LASSO), Ridge, Elastic Net, mRMR, and univariate filtering. Mechanistic validations employed molecular docking, thermal shift assays, surface plasmon resonance (SPR), co-immunoprecipitation, ubiquitination assays, and lipidomics. single-cell RNA sequencing compared malignant osteosarcoma cells with normal bone microenvironment components.

**Results:**

B1 exhibited dose-dependent anti-tumor effects superior to BBR. Machine learning-driven integration of proteomic profiles unanimously nominated Sterol CoA desaturase (SCD) as the key target across all feature selection algorithms, showing both maximal relevance and minimal redundancy. Mechanistically, B1 acts as a molecular glue that recruits the E3 ligase neural precursor cell expressed, developmentally down-regulated 4-like (NEDD4L) to SCD, inducing its ubiquitination and degradation. Single-cell RNA sequencing confirmed significant overexpression of SCD in malignant osteosarcoma cells, further highlighting its therapeutic relevance. Computationally prioritized SCD targeting disrupted lipid metabolism, causing saturated lipid accumulation, mitochondrial damage, and oxidative stress. This ultimately promoted glutathione peroxidase 4 (GPX4)-mediated lipid peroxidation and ferroptosis. Resistance to B1 occurred with SCD overexpression, while arachidonic acid supplementation partially restored tumor survival.

**Conclusions:**

By incorporating machine learning into drug target discovery, we established B1 as a ferroptosis inducer targeting the NEDD4L-SCD axis. Our study provides both a robust therapeutic strategy against chemoresistant osteosarcoma and a compelling blueprint for AI-augmented oncology drug development.

**Graphical Abstract:**

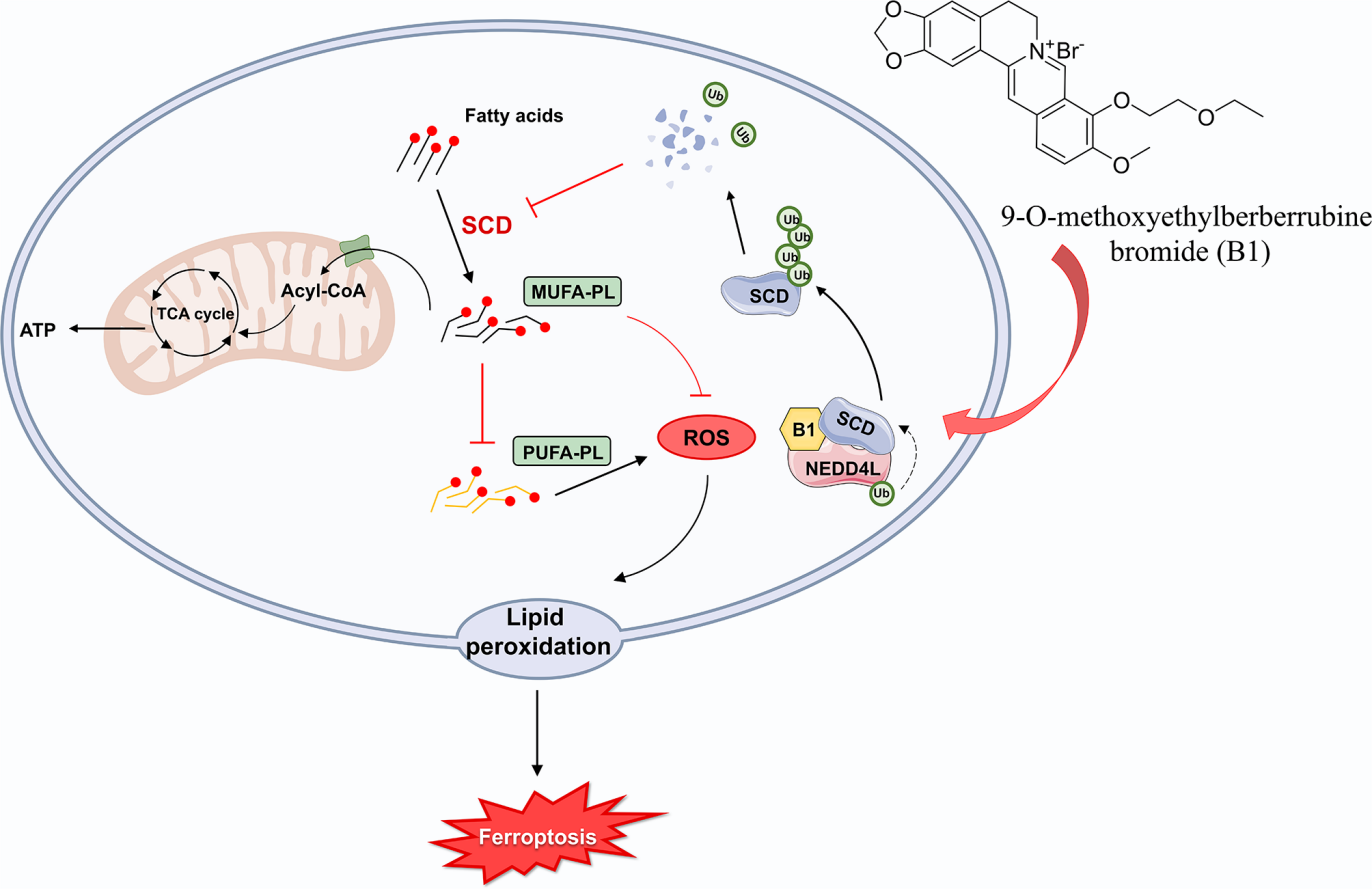

**Supplementary Information:**

The online version contains supplementary material available at 10.1186/s12967-025-07358-6.

## Background

Osteosarcoma (OS), a primary malignant tumor originating in the bone marrow, poses a significant health threat to young adults aged 10 to 25 years [1]. Over the past 30 years, improvements in patient survival rates for osteosarcoma have been largely incremental [2]. Currently, the MAP regimen, which includes high-dose methotrexate, cisplatin, and doxorubicin, is the standard first-line treatment for patients diagnosed with osteosarcoma [3]. As treatment strategies evolve, the combination of neoadjuvant chemotherapy and surgical intervention has contributed to an enhanced overall survival rate. However, clinical outcomes for osteosarcoma patients have not demonstrated substantial improvements in recent decades. Additionally, chemotherapy resistance is a prevalent issue, with the 5-year survival rate remaining below 70% ^2^. Consequently, addressing chemotherapy resistance and developing novel therapeutic agents for osteosarcoma are critical to improving treatment efficacy. Berberine (BBR), an alkaloid derived from the traditional Chinese medicinal plant Coptis chinensis, possesses various pharmacological properties, such as antidiarrheal [4], antibacterial [5], anti-hypertensive [6]. Recent studies have suggested that BBR may act as a potent anti-tumor agent in various cancers [7]. Notably, BBR derivatives have shown enhanced potency as antitumor agents, exhibiting greater inhibition of cell proliferation and increased apoptosis-inducing activity compared to their parent compound, BBR [8], suggesting that berberine derivatives could serve as promising candidates for tumor chemotherapy.

Recent studies have demonstrated that both fatty acid synthesis and glycolysis are crucial for the energy supply in cancer cells [9]. Fatty acids play a crucial role in the synthesis of phospholipids in cancer cell membranes and contribute to the activation of important signaling pathways [10]. In terms of energy production, cancer cells predominantly depend on ATP generated via fatty acid β-oxidation to satisfy their energy needs, while also utilizing nicotinamide adenine dinucleotide phosphate (NADPH) to preserve redox homeostasis [11, 12]. SCD is an enzyme involved in lipid modification, and its expression is often elevated in various cancers, including ovarian, liver, and breast cancers [13–15]. This enzyme facilitates the conversion of saturated fatty acids (SFAs) to monounsaturated fatty acids (MUFAs). Notably, the inhibition of SCD activity, particularly under conditions of limited exogenous lipids, can significantly impede tumor cell proliferation [16]. Compared to polyunsaturated fatty acids (PUFAs), MUFAs exhibit greater resistance to peroxidation, thereby mitigating lipid peroxidation and ferroptosis. The inactivation of MUFAs peroxidase synthetases, such as SCD, renders cancer cells more vulnerable to ferroptosis [17]. Research indicates that SCD can shield tumor cells from ferroptosis induced by lipid peroxidation through the action of Coenzyme Q10 ^18^. The influence of SCD on osteosarcoma progression through the regulation of fatty acid metabolism in osteosarcoma cells has yet to be explored. Considering the critical role of SCD in lipid metabolism and ferroptosis within tumor cells, discovering potent SCD inhibitors could offer a promising approach for treating osteosarcoma.

Machine learning has revolutionized the field of drug discovery by providing powerful computational approaches to analyze complex biological data and identify critical therapeutic targets [18]. By leveraging algorithms capable of handling high-dimensional omics data, researchers can now uncover subtle patterns and interactions that traditional methods often overlook. Techniques such as feature selection, classifier training, and predictive modeling have greatly enhanced the efficiency and accuracy of target identification, offering a data-driven path to understanding drug mechanisms and disease pathways [18, 19]. For example, machine learning has enabled drug repurposing for tuberculosis treatment through virtual screening [20], and facilitated precise structural refinement of protein-drug complexes using machine learning-enhanced quantum mechanics [21]. These advances underscore the growing role of machine learning in improving the precision and scalability of drug discovery.

In this study, we applied integrated machine learning strategies to discover the molecular target of a novel berberine derivative, 9-O-methoxyethylberberrubine bromide (B1), and validate its anti-tumor function. Combining proteomic profiling with multi-algorithm feature selection, we identified sterol-CoA desaturase (SCD) as the primary target of B1. Mechanistically, B1 acts as a molecular glue that recruits the E3 ligase NEDD4L to SCD, inducing its ubiquitination and degradation and ultimately triggering ferroptosis in osteosarcoma cells. Our results underscore the utility of machine learning in facilitating targeted cancer therapy and provide a strong rationale for the further development of B1 as a treatment for osteosarcoma.

## Methods

### In vivo growth and metastasis assays

In the in vivo tumor growth experiments, BALB/c nude mice or NYG mice were subcutaneously injected with 1 × 10⁶ treated cells in the right axilla to establish the subcutaneous model. For the orthotopic model, 5 × 10⁵ cells were introduced into the tibial bone cavity of female BALB/c nude mice. The subjects were aged between four and six weeks at the beginning of the study. All procedures complied with NIH guidelines and were approved by the Animal Care Committee of Harbin Medical University (Ethics Approval No. IRB3043723).

### Cell culture and treatment

Human osteosarcoma cell lines U2OS and 143B, along with the HEK293T cell line, were obtained from FuHeng Cell Center, Shanghai. All cell lines were cultivated in DMEM supplemented with 10% fetal bovine serum (FBS) (Biological Industries, Israel). The human bone marrow mesenchymal stem cells (hBMSCs) were from Cyagen Biosciences (China). The HOS cell line was grown in MEM medium containing 10% FBS. All cultures were maintained at 37 °C in a 5% CO₂ atmosphere. Additionally, each cell line was tested for mycoplasma contamination to ensure clean cultures, and all were authenticated by short tandem repeat (STR) profiling by their respective providers.

### Cells transfection

Cells were transfected with plasmids HA-SCD (GeneChem, Shanghai), FLAG-NEDD4L, Myc-Ub, Myc-K48-Ub, and Myc-K63-Ub (Obio Technology, Shanghai) using Lipofectamine™ 3000 (Invitrogen, USA). Twenty-four hours after transfection, experiments were initiated. The specific siRNA sequence targeting SCD was 5’-AAUGAUCAGAAAGAGCCGUAG-3’. The SCD plasmid was manufactured by Jikai Gene Company in Shanghai. Additionally, lentiviral vectors HBLV-h-SCD-HA-LUC-PURO (Lv-SCD) and HBLV-LUC-PURO (Lv-NC) were produced by HANBIO (China).

### Hematoxylin and eosin (H&E) staining

After the mice were euthanized, tumors were collected and fixed in 4% paraformaldehyde (PFA) for 24 h. The tissues were then paraffin-embedded and sectioned into 4-micrometer thick slices. H&E staining was performed using the Solarbio kit (Catalog No. G1120). The stained sections were observed under a confocal microscope, and images were analyzed using LAS V4.13 software (Leica Microsystems, Germany).

### Immunochemistry staining

Paraffin-embedded tumor sections underwent deparaffinization and antigen retrieval before being incubated overnight at 4 °C with primary antibodies targeting Ki67 (1:2000, ABclonal, A13309) and SCD (1:200, ABclonal, A16429). Following primary antibody incubation, sections were treated with secondary antibodies, then stained with DAB (ORIGEN, ZLI-9018) and hematoxylin (Solarbio). After dehydration and mounting with resin, the slides were examined under a Leica Microsystems microscope. Protein expression levels were quantified using Image J and Visiopharm software.

### Terminal dUTP nick-end labeling (Tunel) assay

Apoptosis was measured by applying the Tunel assay kit (Roche, In Situ Cell Death Detection Kit, POD 11684817910) to frozen sections of tumor tissues or cultured cells. The stained samples were visualized using an Olympus FV1000 microscope, photographed, and the resulting images were quantitatively analyzed with Image J software.

### Cell counting kit-8 assay (CCK-8)

After treatment under specific conditions, 100 µL of complete culture medium and 10 µL of CCK-8 reagent (MCE, cat NO HY-K0301) were added to each well of a 96-well plate, and the absorbance was measured at 450 nm (TECAN INFINITE 200).

### Ethynyl-2-deoxyuridine (EdU) staining assay

Human osteosarcoma cells were seeded at a density of 2.0 × 10⁵ cells per dish and incubated with 30 µM EdU (RiboBio) for 90 min. After the incubation, the cells were fixed with 4% formaldehyde and permeabilized using 0.5% Triton X-100. Following staining with Apollo reagent and DAPI, the samples were observed under an OLYMPUS FV1000 confocal microscope. The percentage of EdU-positive cells (EdU index %) was calculated using Image-Pro Plus software.

### Colony-formation assay

For the colony formation assay, 1,000 cells were plated in six-well plates and cultured at 37 °C in a 5% CO₂ incubator for seven days to allow colony growth. On day seven, the colonies were fixed by immersing the wells in 100% methanol for 20 min. After fixation, the colonies were stained with a 0.1% crystal violet solution for 30 min. Once dried, the colonies were counted.

### Migration assay

Human osteosarcoma cells were plated in six-well plates at a density of 2.5 × 10⁵ cells per milliliter. Once the cell layer achieved approximately 70% confluence, a wound was introduced by making a scratch with a 200 µL pipette tip. After rinsing the cells with PBS, treatments were applied using DMSO, varying concentrations of B1, and Doxorubicin. Photographs of the scratch area were taken immediately after wounding and again after 24 h using a Nikon ECLIPSE TS100 light microscope. The extent of wound closure was quantified utilizing ImageJ software from the National Institutes of Health (NIH).

### Invasion assay

To evaluate cell invasion, a 24 mm Transwell^®^ chamber (Corning #3412, USA) was employed following established procedures. A total of 5 × 10⁴ cells treated with B1 or Dox were suspended in 200 µL of serum-free DMEM and added to the upper chamber. The lower chamber was filled with DMEM supplemented with 10% fetal bovine serum (FBS). After a 24-hour incubation, cells that passed through the membrane were stained with 0.1% crystal violet (Beyotime Biotechnology, China) for 15 min. The stained cells were then counted using a Nikon ECLIPSE TS100 light microscope.

### Cellular Thermal Shift Assay (CETSA)

After trypsinization, cells were quickly frozen in liquid nitrogen for 3 min, then thawed three times. The lysates were centrifuged at 20,000 g for 20 min at 4 °C, and the supernatant was collected. For treatment, 50 µg/mL B1 was added to one group, while an equal volume of deionized water was added to another. These samples were then exposed to heat shock at temperatures of 44 °C, 48 °C, 52 °C, 56 °C, 60 °C, 64 °C, 68 °C, and 72 °C for 3 min each, followed by cooling to room temperature for an additional 3 min. Finally, Western blot analysis was conducted to detect and quantify the protein levels.

### Western blot analysis

Proteins were extracted from tissues or cells using RIPA Lysis Buffer (Beyotime, China) and centrifuged at 13,500 rpm for 15 min at 4 °C to obtain the lysate. Protein concentrations were measured using a BCA Protein Assay Kit (Cat# P0010S; Beyotime). For Western blotting, 100 µg of protein from each sample was electrophoresed, transferred to membranes, and blocked to prevent non-specific binding. The membranes were incubated overnight at 4 °C with primary antibodies, including rabbit anti-SCD (1:1000; Cat# A16429; ABclonal, USA), rabbit anti-GPX4 (1:1000; Cat# A13309; ABclonal, USA), mouse anti-tubulin (1:5000; Cat# AC021; ABclonal, USA), rabbit anti-NEDD4L (1:2000; Cat# 13690-1-AP; ProteinTech, China), rabbit anti-DYKDDDDK tag (1:10000; Cat# 20543-1-AP; ProteinTech, China), and rabbit anti-HA tag (1:10000; Cat# 51064-2-AP; ProteinTech, China). The next day, membranes were treated with secondary antibodies: either monoclonal anti-rabbit IgG (1:5000; Cat# RS23910; ImmunoWay, USA) or monoclonal anti-mouse IgG (1:5000; Cat# RS23920; ImmunoWay, USA) for one hour at room temperature. Protein bands were detected using the Odyssey CLx imaging system, and band intensity was quantified with LI-COR Image Studio Software (LI-COR Biosciences, Lincoln, NE, USA).

### Co-immunoprecipitation assay

Cells were first centrifuged at 3000 rpm for 10 min and then re-suspended in NP-40 lysis buffer containing PMSF and phosphatase inhibitors (Beyotime). The lysates underwent ultrasonic disruption three times to ensure thorough cell breakage. Following this, the samples were centrifuged at 13,500 rpm for 15 min, and the resulting supernatant was collected. This supernatant was incubated overnight at 4 °C with antibodies targeting SCD, HA, DYKDDDDK, and NEDD4L. Protein A/G magnetic beads were subsequently added to the mixture and left to bind overnight at the same temperature. After three washes to remove non-specific bindings, the proteins were eluted by heating the beads in sample loading buffer at 95 °C for 8 min. Finally, the eluted proteins were separated using electrophoresis and analyzed through immunoblotting.

### Tandem mass tag (TMT)-based proteomic analysis

Protein samples underwent enzymatic digestion and desalting before being labeled with the TMT Mass Labeling Kit (ThermoFisher). The labeled peptides were then pooled, dried, and reconstituted. Separation was achieved using strong cation exchange chromatography, followed by an additional desalting and vacuum concentration step. The final peptide solutions were dissolved in 40 µL of 0.1% formic acid and analyzed using a Q-Exactive Mass Spectrometer (ThermoFisher) coupled with Easy nLC. The resulting raw mass spectrometry data were subsequently searched against relevant databases for identification and quantification.

### Molecular modeling

The SCD and NEDD4L proteins were selected as docking templates (source: www.wwpdb.org). The QuickPrep module of Molecular Operating Environment (MOE) 2018 was employed to process the proteins, which involved tasks such as adding hydrogens, calculating charges, removing water molecules, and performing energy minimization. Using this module, the protein was manually docked with the small molecule drug B1.

### Surface plasmon resonance (SPR) assay

The experiments were performed using a Biacore 8 K system (Biacore AB, GE Healthcare) equipped with a CM5 sensor chip (Cytiva). Prior to experimentation, all solutions were filtered through 0.22 μm Millipore filters. The SCD protein was attached to the chip via amine coupling, which involved activating the chip surface with a 1:1 (v/v) mixture of NHS and EDC, followed by the introduction of SCD in an acetate buffer to facilitate binding. Any remaining reactive sites were subsequently blocked with ethanolamine. Compound 1 was prepared in HBS buffer at varying concentrations and introduced to the chip for 180 s, with the dissociation phase also monitored for 180 s. After each injection cycle, the sensor chip was regenerated by applying a glycine/HCl solution. Data acquisition and analysis were conducted using Biacore Insight software (Version 2.0.15.12933).

### RNA extraction

RNA was isolated by lysing cells with Trizol reagent at room temperature. Following this, chloroform was added, and the mixture was vigorously agitated before being centrifuged at 12,000 × g for 15 min at 4 °C. The upper aqueous layer was carefully separated and combined with isopropyl alcohol, ensuring thorough mixing. The solution was then centrifuged again at 12,000 × g for 10 min at 4 °C and left to stand for 30 min, after which the supernatant was removed. The RNA pellet was subsequently washed with 1 mL of 75% ethanol, the excess liquid was drained, and the RNA was dried on filter paper at 4 °C. Finally, the dried RNA was dissolved in 10 µL of DEPC-treated water.

### Quantitative real-time PCR (qRT-PCR)

cDNA was produced using a reverse transcription kit from Thermo Fisher Scientific (Catalog #00676299). Subsequently, quantitative PCR was performed on an Applied Biosystems 7500HT Fast real-time PCR machine utilizing SYBR Green PCR Master Mix from Roche (Catalog #31598800). GAPDH served as the internal reference for normalizing mRNA expression levels. The primer sequences employed are detailed below for both forward (F) and reverse (R) primers (5’-3’).

SCD-F: TTCCTACCTGCAAGTTCTACACC.

SCD-R: CCGAGCTTTGTAAGAGCGGT.

GAPDH-F: AGCCACATCGCTCAGACAC.

GAPDH-R: GCCCAATACGACCAAATCC.

### LC-MS/MS iipidomic analysis

Cellular samples were placed into 2 mL centrifuge tubes and combined with 1 mL of a chloroform-methanol mixture in a 2:1 ratio, along with 100 mg of glass beads. The tubes were then rapidly frozen using liquid nitrogen for 2 to 3 min. After thawing, the samples underwent homogenization at 55 Hz for one minute, a process repeated twice to ensure thorough disruption. The homogenized mixtures were centrifuged at 12,000 rpm for 5 min at 4 °C, and the clear supernatant was carefully collected. This supernatant was then mixed with 2 mL of a 1% methanol sulfate solution and subjected to esterification by heating in an 80 °C water bath for 30 min. Following cooling, 1 mL of n-hexane was added to facilitate lipid extraction, and the samples were centrifuged once more. The lipid-rich supernatant was combined with methyl salicylate as an internal standard, and 200 µL of this solution was transferred into a test vial. Lipidomic analysis was then conducted using HPLC coupled with mass spectrometry (electron ionization). Data were acquired in Selected Ion Monitoring (SIM) mode for precise lipid identification and quantification.

### Machine learning feature selection

To identify robust candidate genes, multiple feature selection methods were applied. LASSO regression with cross-validation was used for sparse modeling, retaining genes with non-zero coefficients. Elastic Net (α = 0.5) and Ridge regression were also employed, with absolute coefficients indicating feature importance. Additionally, minimum Redundancy Maximum Relevance (mRMR) was used to balance relevance and redundancy based on mutual information, while ReliefF evaluated importance by comparing nearest neighbors. For comparison, univariate analysis using absolute t-statistics was conducted to rank genes by group differences. The top three genes from each method were selected, and overlaps were visualized via UpSet plot, highlighting consensus candidates.

### ROS staining and MitoSox staining

Reactive oxygen species (ROS) within cells were quantified using Beyotime’s OxiSelect™ Intracellular ROS Assay Kit (S0033S) following the manufacturer’s instructions. Tumor tissues were sectioned using a cryostat and placed on glass slides, while cells were cultured in 24-well plates or specialized confocal dishes. After fixing the cells and permeabilizing their membranes, they were treated with a DCFH-DA solution diluted 1:1000 in PBS and incubated at 37 °C for 20 min. The resulting fluorescence was captured using either fluorescence or confocal microscopy and analyzed with ImageJ software. To specifically measure mitochondrial ROS, the MitoSOX™ Staining Kit from Invitrogen was utilized. Cells were incubated with the MitoSOX™ reagent in dark conditions for 30 min, after which images were taken and analyzed.

### Lipid peroxidation assay

Cells were seeded in 24-well plates and incubated for 24 h. Both the cell lines and frozen tumor tissue sections were washed with PBS, then treated with 2 µM BODIPY™ 581/591 C11 (Thermo Fisher Scientific, USA) at 37 °C for 30 min. Fluorescent images were then captured using an inverted fluorescence microscope.

### Single-cell RNA sequencing analysis

Single-cell RNA sequencing analysis integrated data from six osteosarcoma samples (GSE162454) and 68 K PBMC controls [22]. Following data normalization and quality control using gene counts, UMI counts, mitochondrial and ribosomal gene content, potential doublets were identified and removed. We performed Harmony-based batch correction using sample and batch covariates followed by UMAP visualization and graph-based clustering at 0.2 resolution. Cell types were annotated through automated classification with reference to the Human Primary Cell Atlas, supplemented by manual refinement using established marker genes. Osteoblast clusters exhibiting abnormal copy number variations relative to immune and endothelial references were designated malignant. These malignant cells were stratified into SCD-positive and SCD-negative subgroups based on expression thresholds. Differential expression analysis identified significant genes between subgroups, visualized as volcano plots, with subsequent functional enrichment (GO/KEGG) and pathway analysis through GSEA using MSigDB Hallmark gene sets.

### ATP assay

ATP concentrations were quantified using the Promega FF2000 ATP assay kit. A series of ATP standards were prepared by performing tenfold serial dilutions from 10 µM to 100 µM. For each measurement, 5 µL of either the ATP standard or the experimental sample was combined with 50 µL of the assay reagent. Luminescence was recorded to establish a standard calibration curve. To measure ATP in the samples, cells were lysed with lysis buffer, and 5 µL of the lysate was mixed with 50 µL of the detection solution. The resulting luminescence values were then compared to the standard curve to determine the ATP levels.

### Nile red staining

A 1 mM Nile Red (MedChemExpress, USA; Cat# 7385-67-3) stock solution was prepared in DMSO and further diluted with PBS to a working concentration of 500 nM. For staining, 143B cells were washed twice with PBS after removal of the culture medium. Subsequently, 1 mL of the Nile Red working solution was added to cover the cells, followed by incubation at room temperature for 10 min. After staining, the cells were rinsed with PBS to remove unbound dye. Images were acquired using an Olympus FV1000 fluorescence microscope.

### Immunofluorescence (IF)

Osteosarcoma cells, numbering 200,000 per glass-bottomed well, were incubated for 24 h to allow attachment and growth. Following this period, the cells underwent two washes with PBS and were subsequently preserved using a 4% paraformaldehyde solution for 15 min. To enable antibody access, the cells were treated with 0.3% Triton X-100 for permeabilization and then blocked with goat serum to reduce non-specific binding. An overnight incubation at 4 °C was performed with a primary antibody targeting SCD (dilution 1:200, ABclonal). For studies requiring the visualization of multiple proteins, primary antibodies against HA and Flag tags were applied at a 1:500 dilution each (ProteinTech). After primary antibody incubation, secondary antibodies conjugated to Alexa Fluor 488 or Alexa Fluor 594 (Invitrogen) were added at a 1:500 dilution. The fluorescent signals were then captured using an Olympus Fv10i confocal microscope for detailed imaging and analysis.

### Flow cytometric analysis

143B cells were prepared at a concentration of 1 × 10⁶ cells/mL and treated with Hoechst 33,342 dye at 37 °C for 7 to 10 min. Following this incubation, the cells were pelleted by centrifugation, the excess dye was discarded, and the cell pellet was subsequently stained with propidium iodide (PI) at 4 °C for 15 min in the absence of light. The stained cells were then passed through a 400-mesh filter and subjected to analysis on a CytoFLEX flow cytometer. The staining reagents utilized included Alexa Fluor 488 diluted at 1:500 (Invitrogen) and the FITC Annexin V Apoptosis Detection Kit I (BD Biosciences, Cat# 556547).

### Statistical analysis

Statistical evaluations were conducted using one-way ANOVA for analyzing multiple groups and the Student’s t-test for comparing two groups. Prism 9 software facilitated the data analysis, and results are presented as mean values with their standard error of the mean (mean ± SEM). Each experiment was independently repeated at least three times to ensure reliability. Significance was determined for P values below 0.05, with the following indicators: *P < 0.05; **P < 0.01; ***P < 0.001.

## Results

### B1 exerts excellent anti-tumor efficacy both in vivo and in vitro

**Fig. 1:**
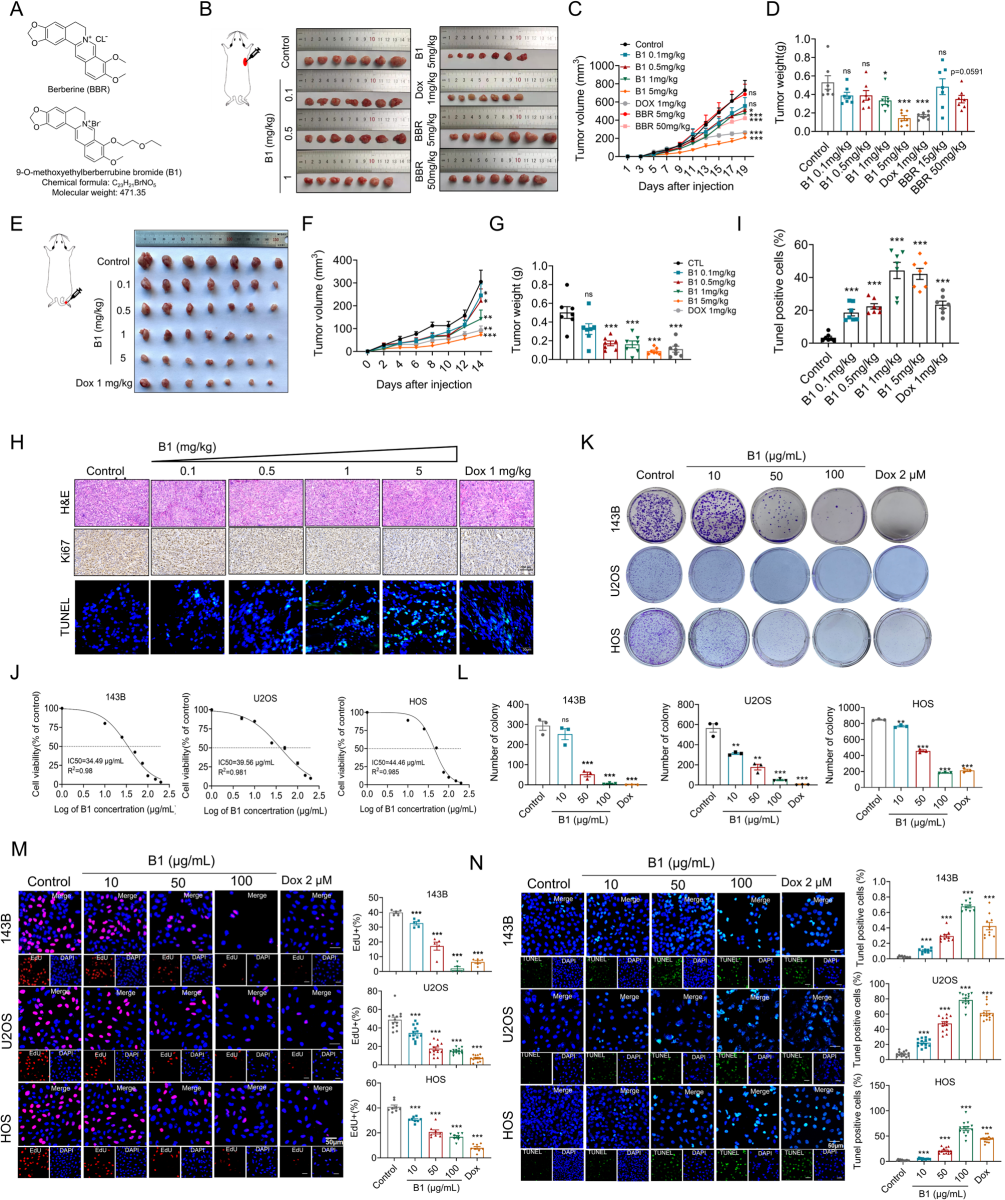
B1 inhibits the growth of osteosarcoma in vivo and in vitro. (**A**) The chemical structures of Berberine (BBR) and its reductive form, 9-O-etherberberrubine bromide (B1), are presented. (**B**) Nude mice bearing subcutaneous tumors derived from the 143B cell line were treated with varying doses of B1 (0.1, 0.5, 1, 5 mg/kg), Doxorubicin (Dox) at 1 mg/kg, and BBR at 5 and 50 mg/kg. Tumor photographs were taken, and tumors were excised on day 19 post-administration. (**C**, **D**) Statistical diagrams illustrate the tumor volume and tumor weight. (**E**, **F**, **G**) Photographs of tumors from 143B tumor-bearing mice are shown after 18 days of daily administration of B1 (0.1, 0.5, 1, 5 mg/kg, via intragastric route), along with thetumor volume, and tumor weight of the mice. *n* = 7. (**H**, **I**) H&E staining, Ki-67 immunohistochemical staining, and TUNEL staining were utilized to assess the proliferation and apoptosis indices of osteosarcoma tissue. *n* = 7. (**J**) Cell viability assays were conducted on various cell lines (143B, U2OS, and HOS) following the administration of different concentrations of B1, *n* = 5. (**K**, **L**) Clone formation assays demonstrated the cell proliferation ability under the treatment of B1 or Dox for 24 h, *n* = 3. (**M**) 143B, U2OS and HOS cells treated different concentrations of B1 or 2 µM positive control drug DOX for 24 h. DAPI and EdU staining patterns of 143B (*n* = 6), U2OS (*n* = 13), and HOS (*n* = 8) cell lines. (**N**) TUNEL staining of 143B (*n* = 10), U2OS (*n* = 12), and HOS (*n* = 12) cell lines. Scale bar: 50 μm. Data are expressed as mean ± SEM. Statistical significance is denoted as follows: ns indicates no significant difference; *P < 0.05; **P < 0.01; ***P < 0.001

Building on previous research indicating that modifications to berberine at the C9 position typically enhance its antitumor activities [23, 24], we synthesized 9-O-methoxyethylberberrubine bromide (B1) (Fig. 1A). To assess the anti-cancer effects of B1 in osteosarcoma and compare it with berberine (BBR), nude mice with 143B cell-induced xenograft tumors were treated daily for 19 days with B1 (0.1, 0.5, 1, 5 mg/kg, i.g.) or BBR (5, 50 mg/kg, i.g.). Doxorubicin (Dox) was used as a positive control (1 mg/kg, i.p.). B1 treatment showed a dose-dependent inhibition of tumor growth, with 5 mg/kg B1 demonstrating an efficacy similar to 1 mg/kg Dox, without affecting body weight (Fig. 1B and D). Importantly, B1 outperformed both BBR groups in tumor suppression (Fig. 1B and D). To better represent the clinical scenario, Balb/c nude mice with 143B cell-driven orthotopic osteosarcoma models were treated with B1 (0.1, 0.5, 1, 5 mg/kg, i.g.) or Dox (1 mg/kg, i.p.) daily for 14 days, showing consistent results. Notably, 0.5 mg/kg B1 had a significant anti-tumor effect, with 1 mg/kg B1 and 1 mg/kg Dox yielding similar results. Additionally, 5 mg/kg B1 proved more effective than Dox in osteosarcoma-bearing mice (Fig. 1E and G). H&E staining revealed substantial tumor necrosis in B1-treated mice, contrasting with controls (Fig. 1H). B1-treated mice also exhibited reduced Ki-67 expression and a higher number of Tunel-positive cells in tumor tissues compared to controls (Fig. 1H and I).

New anti-tumor drugs have diverse mechanisms of action, encompassing the destruction of cancer cells, inhibition of cell proliferation, and prevention of cancer cell dissemination [25]. To assess the impact of B1 on human osteosarcoma (OS) cells, the 143B, U2OS, and HOS cell lines were treated with B1 for 24 h. Cell viability was measured using the CCK8 assay, and the half maximal inhibitory concentration (IC50) was calculated for each cell line (Fig. 1J). Significant reductions in cell viability were observed, with 143B cells showing the greatest sensitivity to B1. Based on these results, three B1 concentrations (10, 50, and 100 µg/mL) were chosen for further analysis, including EdU staining, colony formation, and TUNEL assays. These tests revealed a dose-dependent decrease in colony formation in OS cells treated with B1 (Fig. 1K and L). B1 treatment also inhibited OS cell proliferation in both time- and dose-dependent manners (Fig. 1M). Additionally, the apoptosis rate increased in a dose-dependent fashion following B1 treatment (Fig. 1N). Moreover, B1 significantly suppressed the migration and invasion of all three OS cell lines in a dose-dependent manner (Fig. S1A and S1B), indicating its anti-migratory and anti-invasive effects on tumor cells. These results collectively suggest that B1 exhibits antitumor effects similar to those of Dox in both in vitro and in vivo models.

### Integrated proteomic and machine learning analyses identify SCD as a critical target of B1

**Fig. 2:**
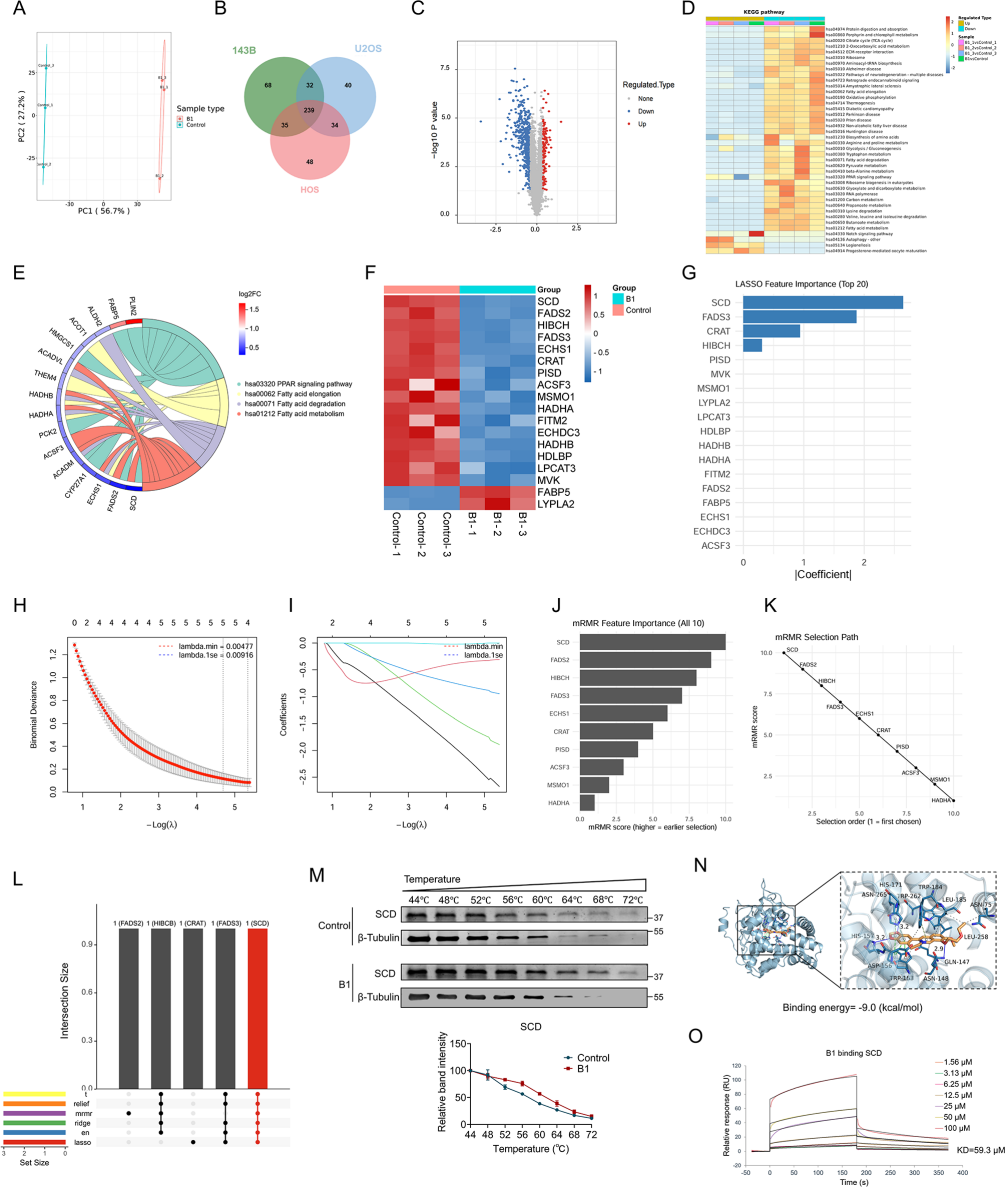
Identification of SCD as a direct target of B1 via integrative proteomics, machine learning, and biochemical validation. (**A**) Principal component analysis (PCA) was performed on quantitative proteomics samples to assess the repeatability of protein quantification, *n* = 3. (**B**) The overlap of proteins with lower expression, as determined by quantitative mass spectrometry-based proteomics, was analyzed among 143B, U2OS, and HOS cells. (**C**) The volcano plot of quantitative proteomics illustrates the differentially expressed proteins in the B1 treatment group compared to the control group, *n* = 3. (**D**) Hierarchical clustering was conducted on KEGG-related pathways, and a heatmap was generated, *n* = 3. (**E**) The chord diagram of KEGG enrichment analysis shows the association between differentially expressed genes and pathways related to fatty acid metabolism. (**F**) The heatmap displays the protein expression changes in the lipid transport and metabolism pathway with the treatment of B1, *n* = 3. (**G**) Variable importance score plot showed the contribution of the candidate proteins in the LASSO diagnostic model. (**H** and **I**) Based on TMT-based proteomics, the Lasso regression analysis and the partial likelihood deviance for prognostic genes are shown. The vertical dashed lines are drawn at the optimal values by the minimum criteria and the 1-standard error (1-SE) criteria. (**J**) Top 10 genes selected by the minimum Redundancy Maximum Relevance (mRMR) algorithm, ranked by their importance scores. (**K**) Feature selection curve of the mRMR algorithm. The performance of a model using an increasing number of top features. The top 10 feature set is highlighted. (**L**) UpSet plot displaying overlaps of feature sets identified by five selection methods. (**M**) The CESTA assay evaluated the interaction between SCD protein and B1 in 143B cells, *n* = 3. (**N**) The binding model between B1 and SCD was generated using AutoDock, with the left panel showing an overall view of the SCD structure and the right panel providing a focused view of B1 in the binding site (SCD in blue, B1 in yellow). (**O**) Surface plasmon resonance (SPR) analysis assessed the interactions of SCD and B1. Data are presented as mean ± SEM. ns: no significant difference; **P < 0.01; ***P < 0.001

With the above studies demonstrating the role of B1 in osteosarcoma development, next, we sought to investigate the underlying mechanism. The altered protein expression in OS cells including 143B, U2OS, and HOS cell lines with or without the treatment of B1 were identified by quantitative proteomic and bioinformatic analysis. PCA analysis shows a distinct separation between the control and B1 group samples (Fig. 2A). There are a considerable number of differentially expressed genes, 239 of which were identical in the three cell lines (Fig. 2B). Figure 2C shows a volcano plot of protein changes after B1 treatment. Kyoto Encyclopedia of Genes and Genomes (KEGG) pathway analysis demonstrated significant enrichment in fatty acid metabolism, PPAR signaling, and energy metabolism pathways (Fig. 2D). The chord diagram specifically visualizes the strong association between the differentially expressed genes and these core fatty acid metabolism-related pathways (Fig. 2E). Beyond these, we also observed alterations in other processes including amino acid metabolism and mitochondrial function, suggesting broad metabolic disruption (as indicated in the full enrichment analysis, Fig. 2D). Ultimately, as a crucial gene in fatty acid metabolism, SCD was the most significantly down-regulated protein, identifying it as a potential target of B1 (Fig. 2F).

To objectively identify the most critical target of B1 from the proteomic data, we employed six distinct machine learning algorithms (t-test, Relief, Mirror, Ridge, Elastic Net, and LASSO) for feature selection. The variable importance plot from the LASSO diagnostic model consistently ranked SCD as the top contributor (Fig. 2G). The optimal lambda values for the prognostic model were determined by minimum criteria and 1-standard-error criteria (Fig. 2H and I). Furthermore, the minimum Redundancy Maximum Relevance (mRMR) algorithm also identified SCD as the top-ranked feature (Fig. 2J), with the feature selection curve confirming the robustness of the top 10 gene set (Fig. 2K). Notably, an UpSet plot analysis revealed that SCD was the only protein unanimously selected by all six feature selection methods (Fig. 2L), underscoring its singular importance.

Cellular Thermal Shift Assay (CETSA) by coculturing the target cells and the drug at different temperatures for a certain period and then lysis, the drug-bound protein will be detected because of the improved thermal stability and less prone to denaturation. We validated the interaction between B1 and SCD using CETSA under the same experimental conditions applied in Thermal Proteome Profiling (TPP). The western blot analysis indicated that B1 stabilized SCD across various temperatures (Fig. 2M). To further elucidate the potential mechanisms underlying the interaction between B1 and the SCD protein, we presented visual molecular docking results of B1 with the unique crystal structure of SCD from the Protein Data Bank (PDB) in Fig. 2N. These results demonstrated a strong interaction, characterized by a binding energy of -9.0 kcal/mol. Additionally, surface plasmon resonance (SPR) experiments confirmed that B1 directly interacted with the SCD protein in a positive dose-dependent manner, with an equilibrium dissociation constant (KD) of 59.3 µM (Fig. 2O). Collectively, these convergent results from both computational and experimental approaches definitively identify SCD as the key target protein mediating B1’s effects.

### B1 promotes the ubiquitination and degradation of SCD protein by enhancing the binding of NEDD4L to SCD

**Fig. 3:**
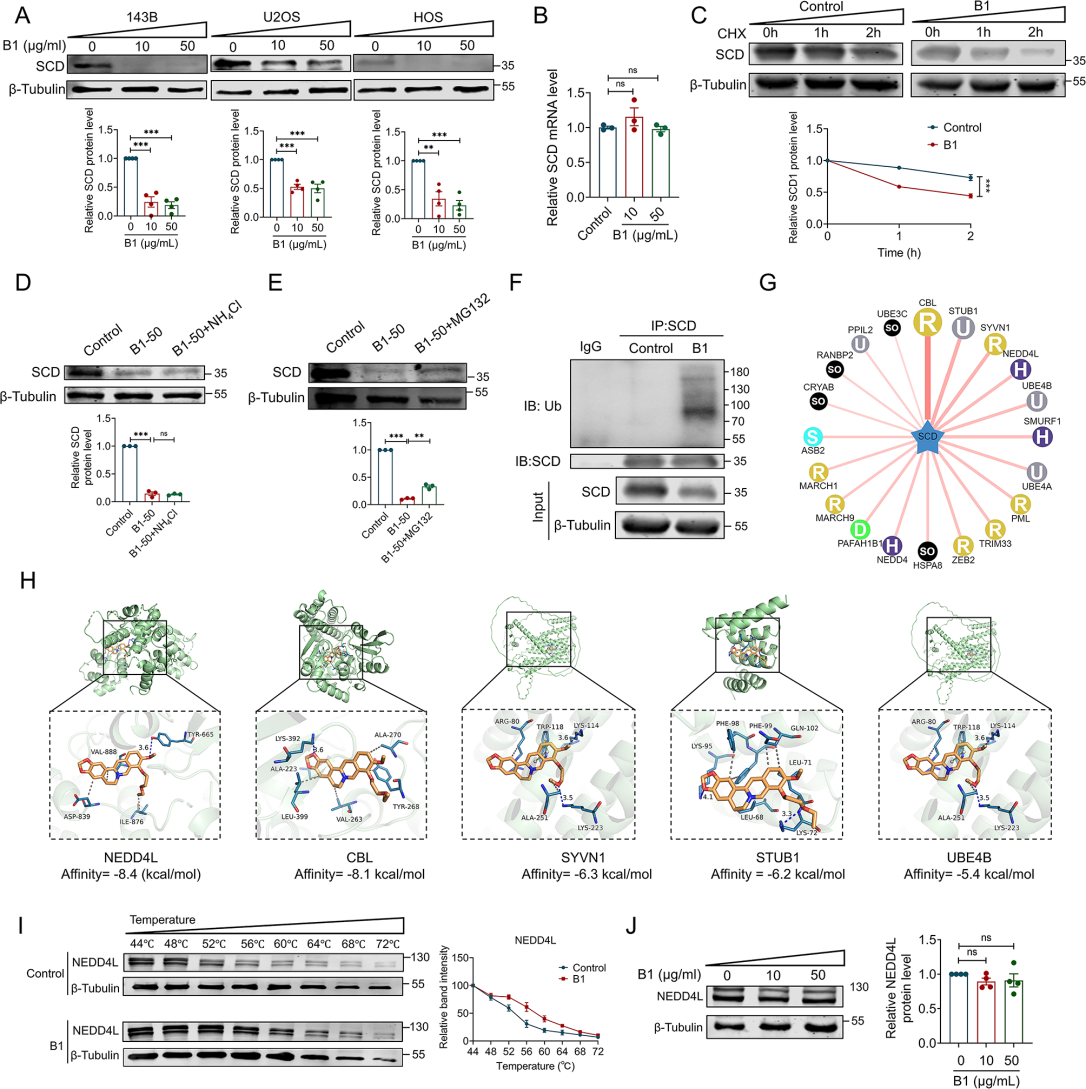
B1 promotes the degradation of SCD mediated by the proteasome pathway. (**A**) The expression levels of SCD protein in 143B, U2OS, and HOS cells were significantly reduced following treatment with 10–50 µg/ml B1 for 24 h, *n* = 4. (**B**) mRNA level of SCD in 143B cells treated with B1. *n* = 3. (**C**) For CHX chase test, 143B cells were treated with CHX 10 µg/ml 24 h after the treatment of B1 (1 µg/ml) and harvested at various times posttreatment (0 h, 1 h, 2 h), *n* = 3. (**D**, **E**) Western blot analysis of SCD protein in 143B cells treated with 10 µg/ml B1, co-treated with 10 mM NH_4_Cl or 5 µM MG132, *n* = 3. (**F**) The ubiquitination of SCD in 143B cells treated with or without 10 µg/ml B1, the cells were treated with MG132 (5 µM) 6 h before collecting samples, *n* = 3. (**G**) Based on the UbiBrowser website, the top 20 E3 ubiquitin ligases of SCD protein were predicted and ranked by score. (**H**) Molecular docking analysis illustrated protein-ligand interactions, utilizing Autodock Vina 1.1.2. The ligand B1 is represented in yellow, and the NEDD4L protein is depicted in green. (**I**) CETSA was used to detect the binding of B1 to NEDD4L protein in 143B cells, *n* = 3. (**I**) Western blot analysis of NEDD4L protein in 143B cells treated with 10–50 µg/ml B1 for 24 h, *n* = 4. Data are expressed as mean ± SEM. ns: no significant difference; **P < 0.01; ***P < 0.001

We conducted maintenance administration of B1 in two additional osteosarcoma cell lines, U2OS and HOS, and observed a significant decrease in SCD protein expression, consistent with findings in the 143B cell line (Fig. 3A). However, quantitative mRNA analysis revealed no significant alteration in SCD transcript levels (Fig. 3B), indicating B1 regulates SCD through post-transcriptional mechanisms. Chlorhexidine (CHX) experiments further demonstrated that B1 facilitates the degradation of SCD protein (Fig. 3C). To elucidate the mechanisms underlying B1-mediated SCD protein degradation, we treated 143B cells with the proteasome inhibitor MG132 and the lysosomal pathway inhibitor NH4Cl, respectively. The results indicated that MG132 significantly reversed the degradation effect of B1 on SCD, while NH4Cl had no impact, suggesting that the inhibition of proteasome activity by B1, rather than lysosomal activity, substantially restored SCD protein levels (Fig. 3D and E). We next examined whether SCD undergoes ubiquitylation for degradation during B1 treatment and confirmed that SCD exhibited ubiquitylation (Fig. 3F). Subsequently, we predicted potential E3 ubiquitin ligases for SCD using the UbiBrowser website (http://ubibrowser.bio-it.cn) (Fig. 3G and Fig. S2) and assessed the binding affinity of B1 to these ligases through molecular docking (Fig. 3H). Our results indicated that among the top five predicted E3 ubiquitin ligases, NEDD4L exhibited the strongest binding affinity to B1, with a binding energy of -8.4 kcal/mol (Fig. 3H). The CETSA experiment further corroborated the interaction between B1 and NEDD4L protein in 143B cells (Fig. 3I). Notably, despite B1 treatment, the protein level of NEDD4L remained unchanged (Fig. 3J). Based on these experimental findings, we hypothesize that B1 may promote the ubiquitination of SCD protein by enhancing the binding of NEDD4L to SCD.

**Fig. 4:**
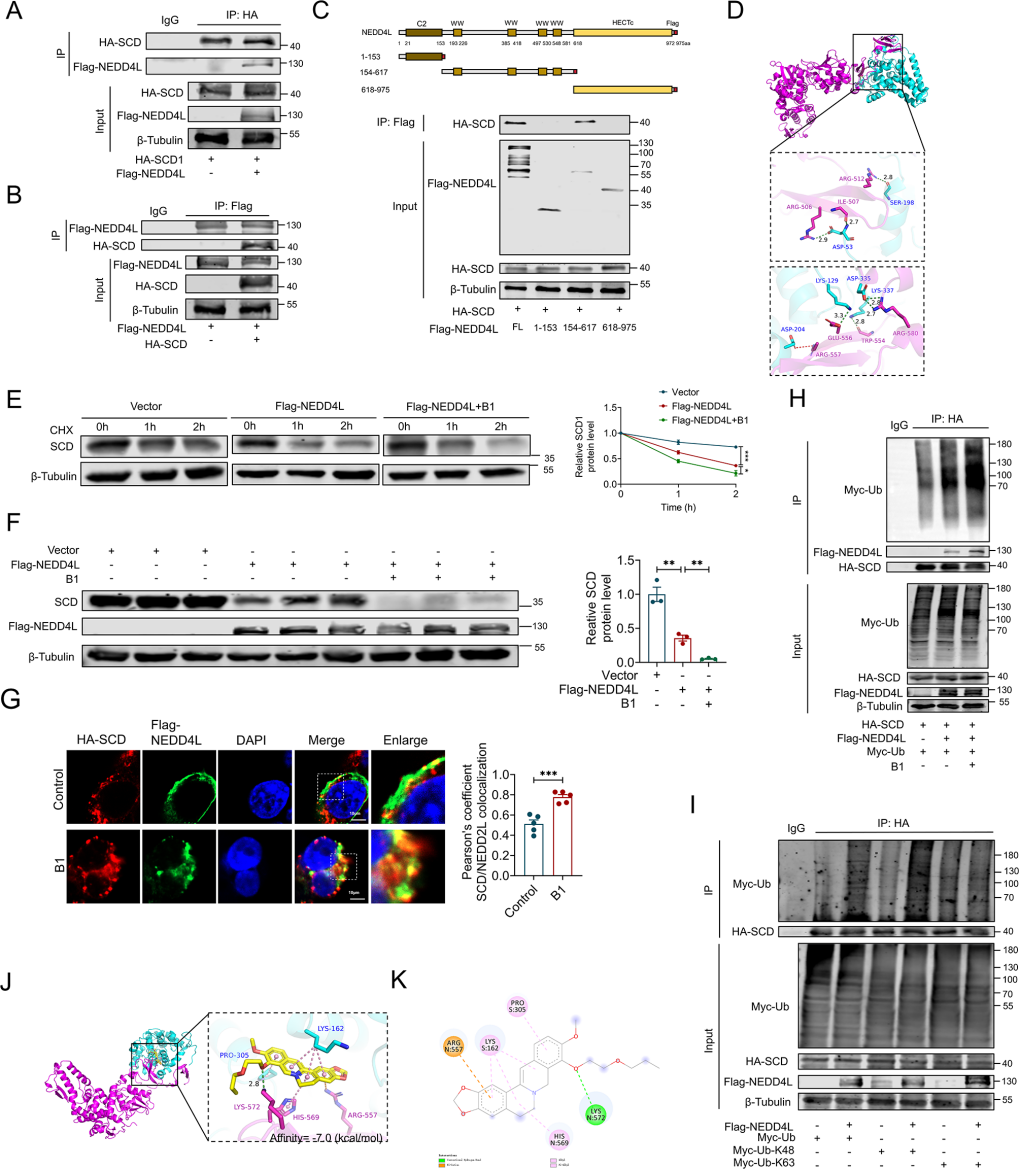
B1 binds to NEDD4L to mediate the degradation of SCD. (**A**, **B**) Co-IP of exogenous SCD and NEDD4L in HEK293T cells after transfection with the indicated plasmids, *n* = 3. (**C**) Co-IP was performed in HEK293T cells after transfection of the SCD full-length plasmid and the truncated plasmid corresponding to NEDD4L, *n* = 3. (**D**) Molecular docking illustrates the three-dimensional binding mode of SCD and NEDD4L protein. (**E**) For CHX chase test, NEDD4L overexpressed143B cells were treated with CHX 10 µg/ml after treating with or without B1 (1 µg/ml) 24 h and harvested at various times posttreatment (0 h, 1 h, 2 h), *n* = 3. (**F**) Western blot analysis of SCD protein in NEDD4L overexpressed 143B cells treated with or without 5 µg/ml B1 for 24 h, *n* = 3. (**G**) Representative images of immunofluorescence colocalization in HEK293T cells. Cells were transfected with HA-SCD, Flag-NEDD4L for 24 h and treated with 10 µg/mL B1 for 2 h before collection. *n* = 5. (**H**) CO-IP results show ubiquitination levels of SCD in HEK293T cells treated with 5 µg/ml B1 and transfected with the indicated plasmids. (**I**) The COIP results show changes in the ubiquitination level of SCD protein in HEK293T cells after cells were transfected with Myc-Ub, Myc-Ub-K48, and Myc-Ub-K63 respectively. (**J**) Molecular docking shows the 3D binding mode of B1 and SCD-NEDD4L protein. Yellow: B1, purple: NEDD4L, blue: SCD. (**K**) The image of 2D interaction showing the binding of the ligand B1 to the SCD-NEDD4L protein. Data are expressed as mean ± SEM. *P < 0.05; **P < 0.01; ***P < 0.001

We conducted co-immunoprecipitation (COIP) experiments to assess the interactions between the NEDD4L and SCD proteins. As anticipated, the COIP results demonstrated that both NEDD4L and SCD proteins could precipitate each other (Fig. 4A and B). Furthermore, we identified that the WW domain of NEDD4L (Δ154–617 aa) is sufficient for binding to SCD (Fig. 4C). Molecular docking studies further confirmed that SCD binds to specific sites within the eight amino acids of the WW domain of NEDD4L (Fig. 4D). Additionally, overexpression of NEDD4L significantly accelerated the degradation rate of SCD protein (Fig. 4E), resulting in a reduced expression level of SCD (Fig. 4F). Concurrently, treatment with B1 further enhanced both the degradation and expression of SCD protein (Fig. 4E and F). Immunofluorescence colocalization studies revealed that four hours post-B1 treatment, the colocalization of SCD and NEDD4L significantly increased, thereby promoting their interaction (Fig. 4G). We also established that NEDD4L has the capacity to ubiquitinate SCD. As shown in Fig. 4H, overexpression of NEDD4L led to a marked increase in SCD ubiquitination. Moreover, B1 treatment further facilitated the interaction between NEDD4L and SCD proteins, resulting in enhanced ubiquitination modification of SCD (Fig. 4H). Further analysis involved the transfection of HEK293T cells with Myc-tagged K48 ubiquitin (K48-Ub) and K63 ubiquitin (K63-Ub) plasmid. The results indicated that only K48-Ub, and not K63-Ub, facilitated the ubiquitination of SCD (Fig. 4I). Importantly, K48-linked ubiquitination is primarily associated with degradation via the proteasome pathway, while K63-linked ubiquitination predominantly contributes to protein stability and signal transduction. Figure 4J and K illustrate the binding mode and two-dimensional interaction profile of B1 with the SCD-NEDD4L protein. The binding energy between B1 and the SCD-NEDD4L complex is -7.0 kcal/mol, suggesting a strong binding affinity. Notably, the ligand establishes a hydrogen bond interaction with LYS572 of the SCD-NEDD4L protein, with a hydrogen bond distance of 2.8 angstroms. Additionally, the hydrophobic functional group of the compound engages in a π-Alkyl/Alkyl conjugation interaction with LYS162, PRO305, and HIS569 of the protein. The benzene ring participates in a π-Cation conjugation interaction with the positively charged ARG557 of the protein. These findings indicate that B1 functions as a molecular glue, enhancing the interaction between NEDD4L and SCD, thereby promoting the ubiquitin-mediated degradation of the SCD protein.

### Depleting of SCD exhibits a tumor suppressor effect in osteosarcoma

**Fig. 5:**
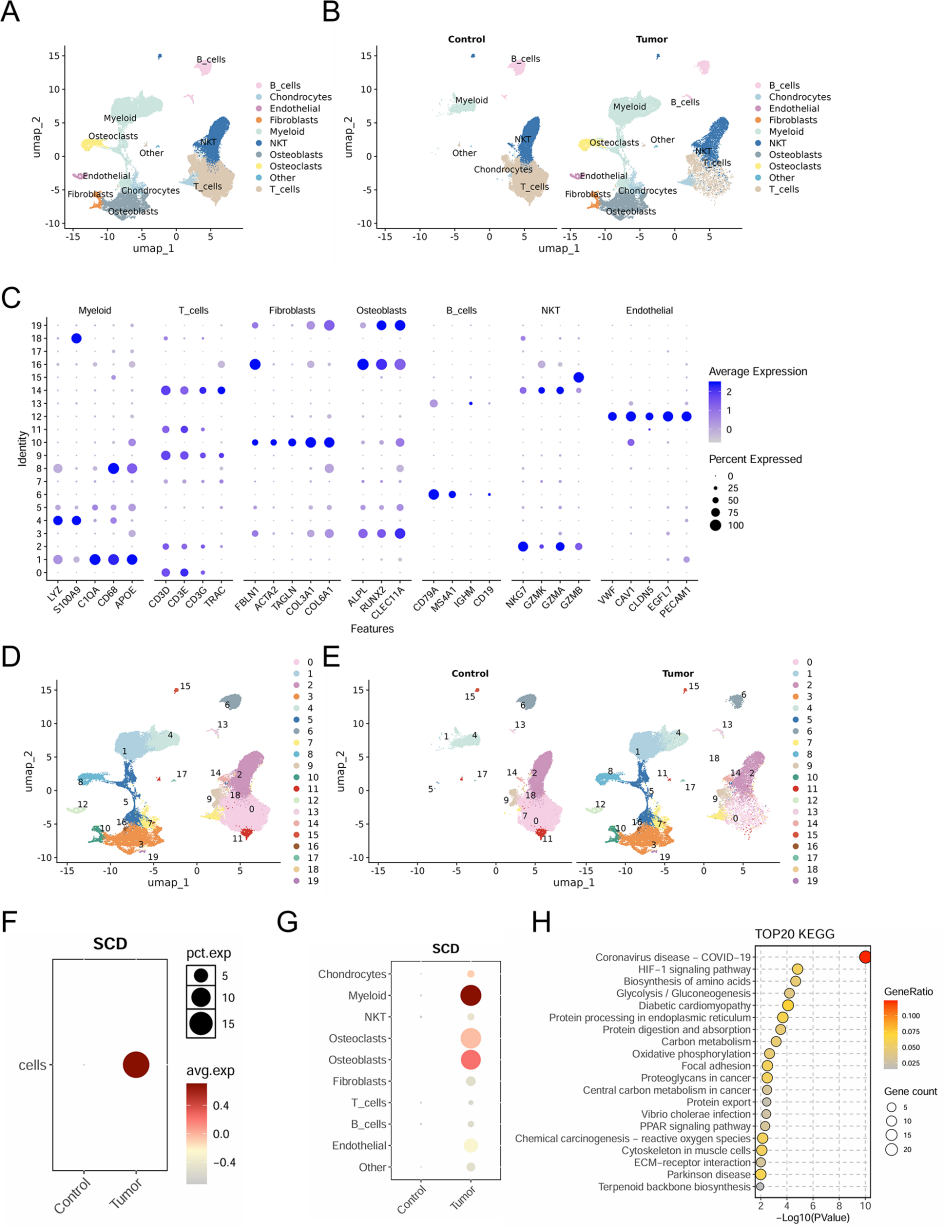
Landscape of SCD expression in single-cell sequencing. (**A**) Global UMAP distribution of cell types in single-cell analysis of osteosarcoma tissues and normal bone tissues. (**B**) Annotated marker genes for major cell types. (**C**) UMAP distribution map grouped by sample (Control group, Tumor group). (**D**) Osteoblasts are further subdivided into 20 subpopulations. (**E**) UMAP distribution map of osteoblast subpopulations grouped by sample. (**F** and **G**) Gene expression characteristics of SCD across different cell types and groups. (**H**) KEGG pathway enrichment analysis of differentially expressed genes (TOP20)

SCD plays a critical role in fatty acid metabolism and has the potential to serve as a novel therapeutic target for various cancers [13, 14]. However, its specific role in osteosarcoma remains poorly understood. To delineate the expression dynamics and functional significance of SCD in osteosarcoma at single-cell resolution, we integrated tumor and bone microenvironment scRNA-seq datasets. Following quality control, dimensionality reduction via t-SNE (Fig. S3A-C) revealed 10 transcriptional clusters based on differentially expressed genes (Fig. 5A and B). Cell-type annotation employed cluster-specific genes validated against canonical biomarkers, with thirty signature gene expressions visualized across clusters (Fig. 5C). Initial assessment demonstrated pronounced batch-driven segregation in raw UMAP projections (Fig. S4), while Harmony integration using sample and batch covariates yielded harmonized cellular distributions consistent with technical artifact mitigation (Fig. S4). Subsequent graph-based clustering (resolution = 0.2) on the corrected manifold identified nineteen biologically distinct subpopulations (Fig. 5D and E). Comparative analysis revealed markedly elevated SCD expression in tumor tissue relative to peripheral blood mononuclear cells (Fig. 5F), with most pronounced enrichment in myeloid cells, osteoclasts, and osteoblasts (Fig. 5G). KEGG pathway analysis of downregulated genes in SCD-deficient malignant cells demonstrated concerted suppression of lipid metabolic and stress-responsive pathways. This signature implies that diminished SCD activity, which compromises fatty acid desaturation, promotes saturated lipid accrual, endoplasmic reticulum stress, and oxidative cascades (Fig. 5H). Thus, we propose this metabolic derangement induces reactive oxygen species overproduction, contributing to neoplastic cellular dysfunction in osteosarcoma.

**Fig. 6:**
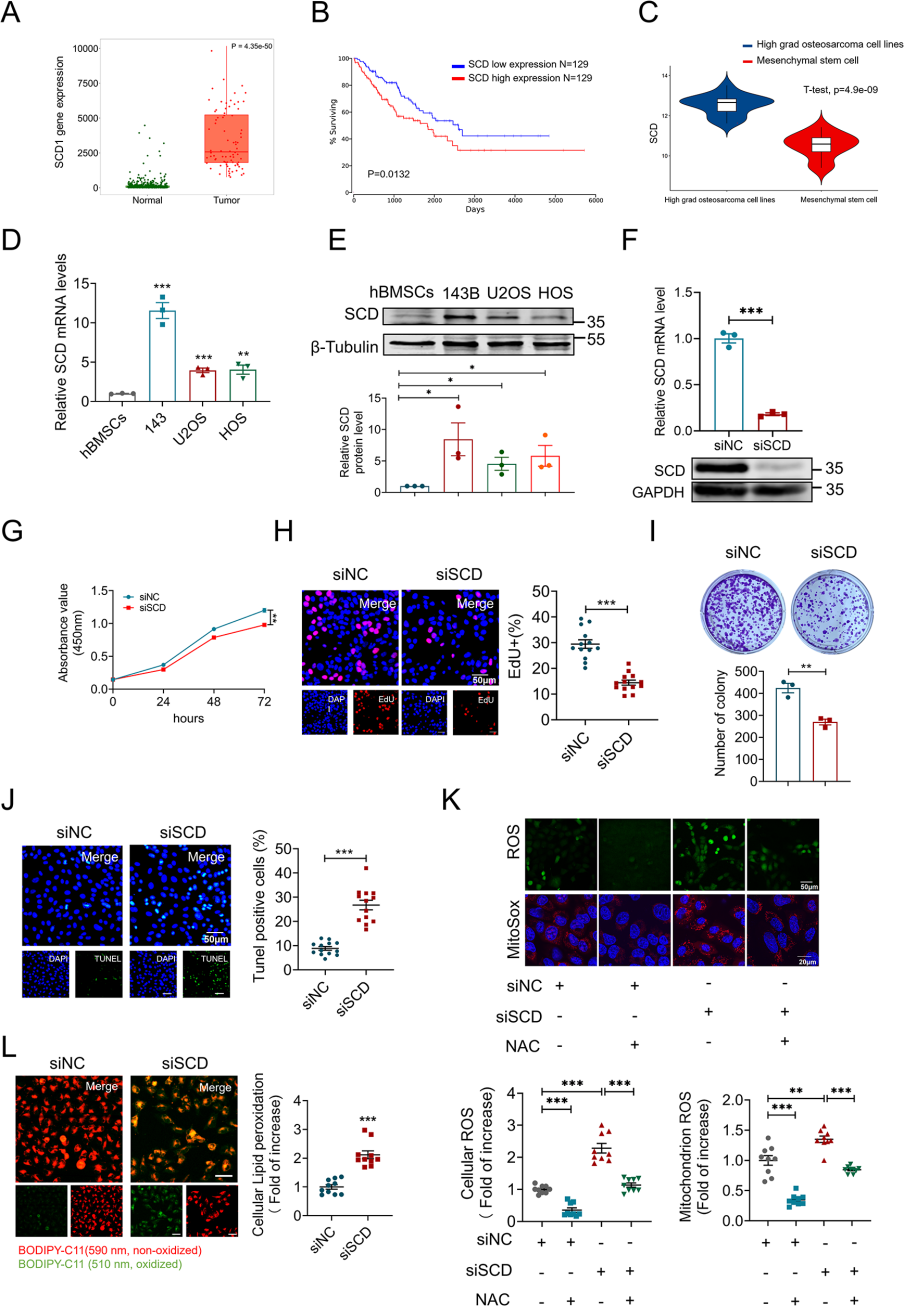
SCD knockdown suppresses osteosarcoma progression. (**A**) TNMplot database was used to compare the gene expression of SCD between normal and tumor tissues. (https://tnmplot.com/analysis/) (**B**) Kaplan-Meier survival curves from TCGA showing longer survival for sarcoma patients with SCD low expression. (**C**) GEO database GEO42352 revealed differential gene expression between high-grade osteosarcoma cell lines and mesenchymal stem cells. (**D**) Relative mRNA levels of SCD in hBMSCs, 143B, U2OS, and HOS (*n* = 3). (**E**) Western blot analysis of SCD protein in 143B, U2OS and HOS compared to hBMSCs (*n* = 3). (**F**-**I**) qRT-PCR and Western blot were used to verify SCD knockdown efficiency (**F**, *n* = 3). CCK8 assay (**G**, *n* = 3), EdU staining (**H**, *n* = 13), and clone formation assay (**I**, *n* = 3) were used to detect the viability and proliferative potential of 143B cells transfected with negative control and SCD siRNA. (**J**) Representative images and statistical diagram of TUNEL staining of 143B cells. The green spots represent TUNEL-positive cells, indicating the apoptotic region. Scale bar: 50 μm (*n* = 13). (**K**) Detection of superoxide in cells and mitochondria using MitoSox and ROS detection probes. Scale bar: 50 μm; *n* = 9. (**L**) The lipid peroxidation of cells transfected with negative control or SCD siRNA was detected by the lipophilic redox-sensitive dye BODIPY 581/591, which shifts its fluorescence from red to green in response to oxidation (*n* = 10). Scale bar: 50 μm. Data were expressed as mean ± SEM. *P < 0.05; **P < 0.01; ***P < 0.001

Consistently, analysis of the TNMplot database confirmed marked SCD upregulation in osteosarcoma tissues relative to matched adjacent normal tissues (Fig. 6A). Furthermore, Kaplan–Meier survival analysis indicated that elevated SCD expression was linked to worse survival outcomes in osteosarcoma patients (Fig. 6B). Bioinformatics analysis also indicated elevated SCD expression in osteosarcoma cells compared to mesenchymal stem cells (MSCs) (Fig. 6C), a finding supported by PCR and western blot results from human bone MSCs (hBMSCs) and human osteosarcoma cell lines, including 143B, U2OS, and HOS (Fig. 6D and E). Next, we performed SCD knockdown in 143B cells (Fig. 6F), and both CCK8 and EdU assays demonstrated that reduced SCD expression significantly decreased cell viability (Fig. 6G and H). Additionally, SCD knockdown severely impaired the colony-forming capacity of 143B cells (Fig. 6I). Notably, depletion of SCD resulted in a significant increase in apoptosis rates in 143B cells (Fig. 6J). Previous studies have shown that downregulation of SCD can induce ferroptosis in cells by promoting abrupt ROS production and lipid oxidation [18, 27]. Reactive oxygen species (ROS) and mitoSOX staining revealed an increase in cellular ROS levels following SCD silencing (Fig. 6K). Additionally, lipid peroxidation staining showed a marked rise in lipid peroxidation levels in 143B cells after SCD knockdown (Fig. 6L). These findings collectively suggest that SCD expression promotes osteosarcoma cell growth by regulating oxidative balance and lipid metabolism.

### Ectopic expression of SCD renders osteosarcoma cells resistant to B1-dependent ferroptosis

**Fig. 7:**
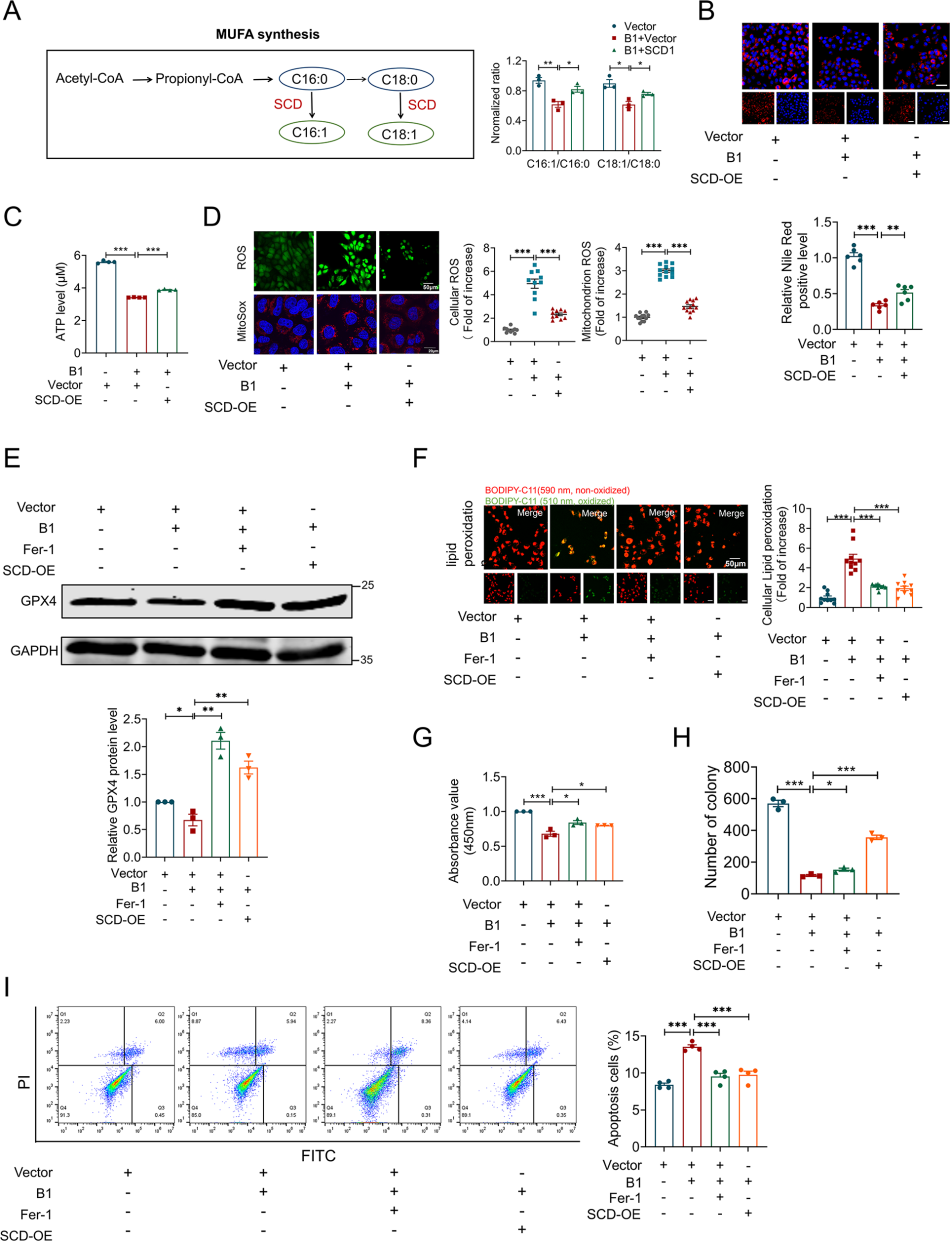
Overexpression of SCD and ferroptosis inhibitor Fer-1 reversed the regulatory effect of B1 on osteosarcoma. (**A**-**D**) 143B cells were exposed to 10 µg/ml B1 for 24 h with or without SCD overexpressing. (**A**) The schematic diagram on the left shows the generation process of monounsaturated fatty acids. GS-MS ratios analysis on the right shows the MUFA/SFA ratios (*n* = 3). (**B**) Nile red staining was used for intracellular lipid droplet analysis, *n* = 6. (**C**) Cellular ATP levels measurement, *n* = 3. (**D**) ROS (*n* = 10) and MitoSox (*n* = 12) detection probes were used to detect intracellular and mitochondrial oxidation levels, respectively. Scale bar: 50 μm. (**E**-**I**) 143B cells were treated with 10 µg/ml B1 for 24 h, and cells were pretreated with 5 µM Fer-1 for 6 h or transfected with SCD plasmid. (**E**) Immunoblot for GPX4 protein (*n* = 3). (F) Confocal imaging of lipid peroxidation staining; Orange, the reduced form of C11-BODIPY; Green, the oxidized form of C11-BODIPY (*n* = 10). (**G**) Cell viability assay (*n* = 3). (**H**) Colony formation experiment (*n* = 3). (**I**) Annexin V-FITC/PI staining assay of apoptotic 143 cells (*n* = 4). Data were expressed as mean ± SEM. *P < 0.05; **P < 0.01; ***P < 0.001

Ectopic expression of SCD confers resistance to B1-induced ferroptosis in osteosarcoma cells. As shown in Fig. 7A, SCD catalyzes the conversion of saturated fatty acids (SFAs), such as stearic acid (18:0) and palmitic acid (16:0), into their monounsaturated forms, oleic acid (18:1) and palmitoleic acid (16:1), via desaturation at the Δ9 position [26]. To explore the impact of B1, with or without SCD overexpression, on fatty acid metabolism, LC/MC analysis was conducted. As shown in Fig. 7A, B1 treatment notably reduced the ratios of C16:1/C16:0 and C18:1/C18:0, an effect that was reversed by SCD overexpression. Additionally, B1 treatment led to reduced lipid accumulation, which was also restored by SCD overexpression (Fig. 7B and Fig. S5). B1 significantly lowered intracellular ATP levels in osteosarcoma cells, but this was reversed by SCD overexpression (Fig. 7C). In line with earlier results, SCD overexpression in osteosarcoma cells diminished the ROS level elevated by B1 treatment (Fig. 7D). GPX4, a key marker for ferroptosis inhibition, plays an essential role in mitigating lipid peroxidation during ferroptosis [27]. B1 treatment notably reduced GPX4 protein levels, a change that could be reversed by either SCD overexpression or the use of the ferroptosis inhibitor Fer-1 (Fig. 7E). As expected, both SCD overexpression and Fer-1 treatment alleviated the increased lipid peroxidation caused by B1 in 143B cells (Fig. 7F), restored cell viability and colony formation (Fig. 7G and H and Fig. S6), and reduced apoptosis (Fig. 7I). Immunofluorescence staining showed that higher B1 doses led to decreased levels of SCD and GPX4 in osteosarcoma tissues (Fig. S7A), while reactive oxygen species (ROS) (Fig. S7B) and lipid peroxidation (Fig. S7C) levels increased in a dose-dependent manner. These results suggest that B1 disrupts fatty acid metabolism by targeting SCD for degradation, leading to the accumulation of ROS and lipid peroxidation, which ultimately triggers ferroptosis in cells.

### The antitumor efficacy of B1 is significantly superior to that of the SCD inhibitor MK-82,455

MK-8245 is a potent SCD inhibitor currently available on the market, recognized for its antidiabetic and anti-dyslipidemic properties, and has demonstrated a certain level of efficacy against liver tumors [28]. Given the strong inhibitory effect of B1 on SCD activity, we aimed to compare the antitumor effects of B1 and MK-8245. We first assessed the IC50 values of MK-8245 in three human osteosarcoma cell lines including 143B, U2OS, and HOS, which were found to be 43.62 µg/mL, 110.4 µg/mL, and 87.26 µg/mL, respectively (Fig. S8A). These values were notably higher than the IC50 values observed for B1 in our previous experiments (Fig. 1J). At a concentration of 50 µg/mL, B1 demonstrated superior effects on the proliferation and colony formation of 143B cells compared to MK-8245, as shown by EdU staining and colony formation assays (Fig. S8B and S8C). Additionally, B1 induced a higher rate of apoptosis in 143B cells compared to MK-8245 (Fig. S8D). These results suggest that B1 is a more potent SCD inhibitor than MK-8245, exerting a stronger inhibitory effect on osteosarcoma cells.

### Arachidonic acid treatment reversed the effects of SCD overexpression, further amplifying the effectiveness of B1

**Fig. 8:**
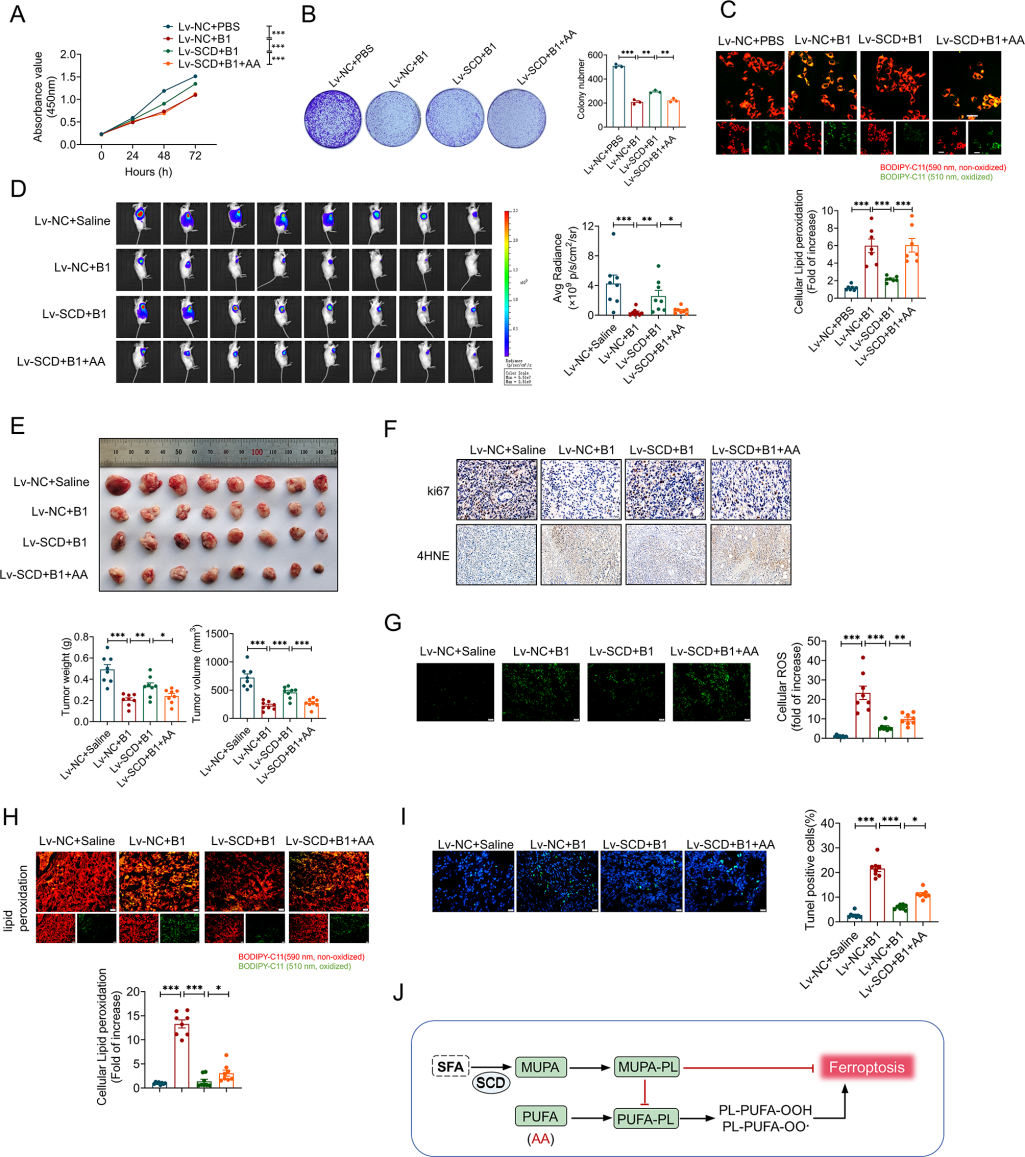
SCD overexpression inhibits the anti-tumor effect of B1, while arachidonic acid exerts a synergistic effect. (**A**-**C**) 143B cells were exposure to 10 µg/ml B1, combing with SCD overexpression or SCD overexpression co-treated with 50 µM arachidonic acid. (**A**) CCK8 assay at 0 h, 24 h, 48 h and 72 h. (**B**) Colony formation experiment (*n* = 3). (**C**) Confocal imaging of lipid peroxidation staining. Orange, the reduced form of C11-BODIPY; Green, the oxidized form of C11-BODIPY (*n* = 7). (**D**) The growth of negative control (Lv-NC) or lentivirus-stable SCD-overexpression (Lv-SCD) 143B cells in mice were monitored through in vivo bioluminescence imaging, and the fluorescence intensity was measured therein, *n* = 8. (**E**) Images of osteosarcoma tissue from mice were presented. Weight and volume of tumors were measured, *n* = 8. (**F**) Immunohistochemical staining revealed the expression levels of Ki67 and 4-HNE in different groups of the collected tumor tissues. (**G**) DCFH-DA fluorescence staining was employed to evaluate the ROS levels in osteosarcoma tissues from subcutaneous tumor-bearing mice (*n* = 6). (**H**) Images of lipid peroxidation staining of frozen sections of the collected tumor tissues. (**I**) TUNEL staining was performed on osteosarcoma tissues, with apoptotic cells shown in green and the nucleus in blue. Scale bar: 50 μm (*n* = 6). (**J**) Mechanism diagram of the regulation of ferroptosis by MUFAs and PUFAs. Data were expressed as mean ± SEM. *P < 0.05; **P < 0.01; ***P < 0.001

Ferroptosis involves a complex balance between oxidative and antioxidative processes. The incorporation of polyunsaturated fatty acids (PUFAs) into polyunsaturated phospholipids (PUFA-PLs) increases the vulnerability of cells to ferroptosis. In contrast, monounsaturated phospholipids (MUFA-PLs) can protect against ferroptosis by reducing the abundance of PUFA-PLs [29]. Arachidonic acid (AA), a widely recognized polyunsaturated fatty acid, has been shown to trigger apoptosis in cancer cells [30]. In vitro experiments using the 143B cell line, which stably overexpresses SCD (Fig S9), demonstrate that AA treatment can counteract the reduction in cell viability caused by SCD overexpression in the presence of B1 (Fig. 8A and B and Fig S11). Notably, SCD overexpression led to a decrease in lipid peroxidation induced by B1, whereas AA treatment exacerbated lipid peroxidation levels (Fig. 8C). To further validate the therapeutic potential of this combination, in vivo experiments were conducted. As illustrated in Fig. 8D and E, the therapeutic efficacy of B1 was significantly diminished in tumor tissues derived from SCD-overexpressing cell lines. However, AA treatment was found to synergistically enhance the therapeutic effect of B1, resulting in a marked reduction in tumor volume and weight (Fig. 8E). Importantly, the synergistic anti-tumor effect of B1 and AA was significantly compromised by SCD overexpression (Fig. 8F). Additionally, SCD overexpression substantially inhibited the production of ROS and the accumulation of lipid peroxidation, thereby suppressing tumor cell apoptosis (Fig. 8G and I). In contrast, AA treatment reversed the effects of SCD overexpression, further amplifying the impact of B1 on cellular oxidative stress and apoptosis (Fig. 8G and I). In conclusion, the novel B1 berberine derivative effectively targets the ubiquitination and degradation of SCD, inhibiting monounsaturated fatty acid synthesis, increasing oxidative stress and lipid peroxidation, and inducing ferroptosis, which collectively suppresses tumor growth (Fig. 8J).

## Discussion

In this research, we developed a new derivative of berberine, 9-O-methoxyethylberberrubine bromide (B1), which we found to be an effective agent in suppressing the aggressive progression of osteosarcoma. This compound acts as a molecular glue by targeting SCD-regulated lipid balance and cellular redox state, thereby inducing ferroptosis in cancer cells. Berberine, an alkaloid isolated from the Chinese herb Coptis chinensis, is known for its broad spectrum of pharmacological effects [31–33]. Increasing evidence supports the anti-tumor effects of berberine across various cancers, including ovarian cancer [34], colorectal carcinoma [35], gastric cancer [36] Notably, berberine derivatives often demonstrate enhanced anti-tumor activity compared to the parent compound, particularly the 9-O-substituted derivatives [23, 24]. In our experiments, we found that a dosage of 0.5 mg/kg B1 produced a significant antitumor effect, surpassing that of berberine at doses of 5 and 50 mg/kg. Furthermore, the efficacy of 1 mg/kg B1 was comparable to that of 1 mg/kg doxorubicin, while 5 mg/kg B1 outperformed doxorubicin in osteosarcoma-bearing mice. Consequently, our findings suggest that the new berberine derivative B1 holds great promise as a potential anticancer drug for osteosarcoma.

The discovery of SCD as B1’s target was critically guided by an integrated machine learning strategy. We employed a combination of LASSO, Ridge, Elastic Net, and mRMR algorithms to analyze our proteomic data. This multi-faceted approach was chosen to leverage their complementary strengths: LASSO for sparse feature selection, Ridge and Elastic Net for handling variable correlation, and mRMR for ensuring feature relevance with minimal redundancy. The unanimous nomination of SCD as the top candidate across all these distinct methods provided exceptional confidence in target identification, far surpassing what any single algorithm could achieve. This robust computational framework demonstrates generalizable utility for prioritizing therapeutic targets in oncology drug discovery.

Molecular glue refers to a class of small molecule compounds that interact with target proteins in a unique manner. Unlike traditional drugs, which typically bind directly to the active site of a target protein, molecular glues modulate protein function and stability by facilitating interactions between distinct proteins [37]. This innovative mechanism offers a novel approach to targeting ‘undruggable’ proteins, significantly broadening the scope and potential applications of drug design. Through screening of compound libraries, researchers identified a molecular glue that enhances the binding of the E3 ligase cereblon to the WIZ protein. This screening also revealed a previously unknown transcriptional repressor protein. The action of the molecular glue leads to the degradation of the transcriptional repressor protein WIZ, thereby activating HbF expression and providing a therapeutic effect for sickle cell anemia [38]. Furthermore, molecular glues circumvent common drug resistance mechanisms by inducing the degradation of target proteins, paving the way for new therapeutic strategies. Lenalidomide, a classic example of a molecular glue drug, promotes the degradation of specific immunomodulatory proteins, such as Ikaros and Aiolos, by binding to CRBN [39]. This mechanism activates the immune system to target cancer cells while evading drug resistance mechanisms associated with genetic mutations or epigenetic changes [40]. In alignment with these findings, our study demonstrates that B1 functions as a molecular glue by enhancing the interaction between the E3 ubiquitin ligase NEDD4L and the SCD protein, facilitating the ubiquitination and subsequent degradation of the SCD protein. While various small molecule drugs have been identified that inhibit SCD expression, B1 represents the first molecular glue specifically targeting SCD protein degradation. This discovery may further advance the development of SCD as a drug target related to tumor treatment.

SCD, also known as 9-fatty acyl-CoA desaturase, is essential for metabolic homeostasis via catalyzing the synthesis of monounsaturated fatty acids, plays a crucial role in maintaining metabolic homeostasis by catalyzing the synthesis of monounsaturated fatty acids (MUFAs) [41]. Metabolic reprogramming is recognized as a significant driver of tumor development, exemplified by the Warburg effect, which results in the production of excess lactate, citrate, and glycerol, subsequently contributing to de novo lipid synthesis through acetyl-CoA carboxylase (ACC) and fatty acid synthase (FAS). Recent studies have revealed that SCD is highly expressed in various malignant tumors, leading to substantial MUFA production [42, 43]. The majority of MUFAs synthesized by SCD are believed to participate in phospholipid biosynthesis, thereby supporting cancer cell growth and metastasis. Quantitative mass spectrometry-based proteomics and bioinformatics analyses have identified that B1 primarily engages in the fatty acid elongation pathway, with SCD being one of the most significantly down-regulated genes. In alignment with previous findings in other tumor types, our results demonstrate that SCD expression is positively correlated with the survival duration of osteosarcoma patients, where its downregulation markedly inhibits osteosarcoma cell proliferation and promotes apoptosis. Importantly, the reduction of SCD protein levels regulated by B1 leads to decreased MUFA synthesis, which results in a diminished ratio of C16:1/C16:0 and C18:1/C18:0. It is well established that MUFAs serve as scavengers of lipid peroxidation [44]. Furthermore, our findings indicate that B1 significantly induces reactive oxygen species (ROS) production, which subsequently results in mitochondrial ROS generation and lipid peroxidation, ultimately disrupting lipid metabolism by targeting SCD.

The loss of cellular redox homeostasis and the consequent production of lipid oxidation are critical factors in the process of cell ferroptosis [45]. Previous studies have established that SCD functions as a suppressor of ferroptosis; its downregulation markedly induces ferroptosis by enhancing reactive oxygen species (ROS) production and lipid oxidation [45–47]. Our research revealed that B1 treatment significantly reduced the protein expression of GPX4, a pivotal protein responsible for limiting lipid peroxides and preventing ferroptosis. Notably, the effects of B1 were reversed upon overexpression of SCD or treatment with Fer-1, a well-known ferroptosis inhibitor. Additionally, we observed that both SCD overexpression and Fer-1 treatment significantly counteracted the promoting effects of B1 on ROS/mitochondrial ROS levels and lipid peroxidation. More importantly, these interventions reversed the inhibition of cell viability, suggesting that a therapeutic strategy involving B1, which inhibits SCD while inducing ferroptosis, may be beneficial in the treatment of osteosarcoma.

## Conclusions

In summary, this study demonstrates that the novel berberine derivative B1 functions as a molecular glue-type degrader by simultaneously engaging the E3 ubiquitin ligase NEDD4L and the substrate SCD protein, thereby facilitating ubiquitin-mediated degradation of SCD. The downregulation of SCD suppresses lipid desaturation, disrupts lipid metabolism, and ultimately induces ferroptosis in osteosarcoma cells, both in vitro and in vivo. These findings provide important insights for the development of new clinical therapeutics against osteosarcoma and highlight the considerable therapeutic potential of C9-modified berberine derivatives.

## Supplementary Information

Below is the link to the electronic supplementary material.

Supplementary Material 1

## Data Availability

The datasets produced and analyzed in this study are not publicly accessible but can be obtained from the corresponding author upon reasonable request.

## Abbreviations

AA: Arachidonic acid
B1: 9-O-methoxyethylberberrubine bromide
BBR: Berberine
CETSA: Cellular thermal shift assay
Dox: Doxorubicin
IC50: Half maximal inhibitory concentration
hBMSCs: Human bone marrow mesenchymal stem cells
LASSO: Least absolute shrinkage and selection operator
MUFAs: Monounsaturated fatty acids
NEDD4L: Neural precursor cell expressed, developmentally down-regulated 4-like
OS: Osteosarcoma
PUFAs: Polyunsaturated fatty acid
SCD: Stearoyl-CoA desaturase-1
SFAs: Saturated fatty acid
