# Supplementary material for: Machine learning-powered discovery of a novel berberine derivative inducing SCD-dependent ferroptosis in osteosarcoma
Source: J Transl Med. 2025 Nov 20;23:1328. doi: 10.1186/s12967-025-07358-6 (PMC12636167; doi:10.1186/s12967-025-07358-6)
Supplement: Supplementary file 1 — Supplementary Material 1 [file 12967_2025_7358_MOESM1_ESM.docx]

**Figure S1**

**
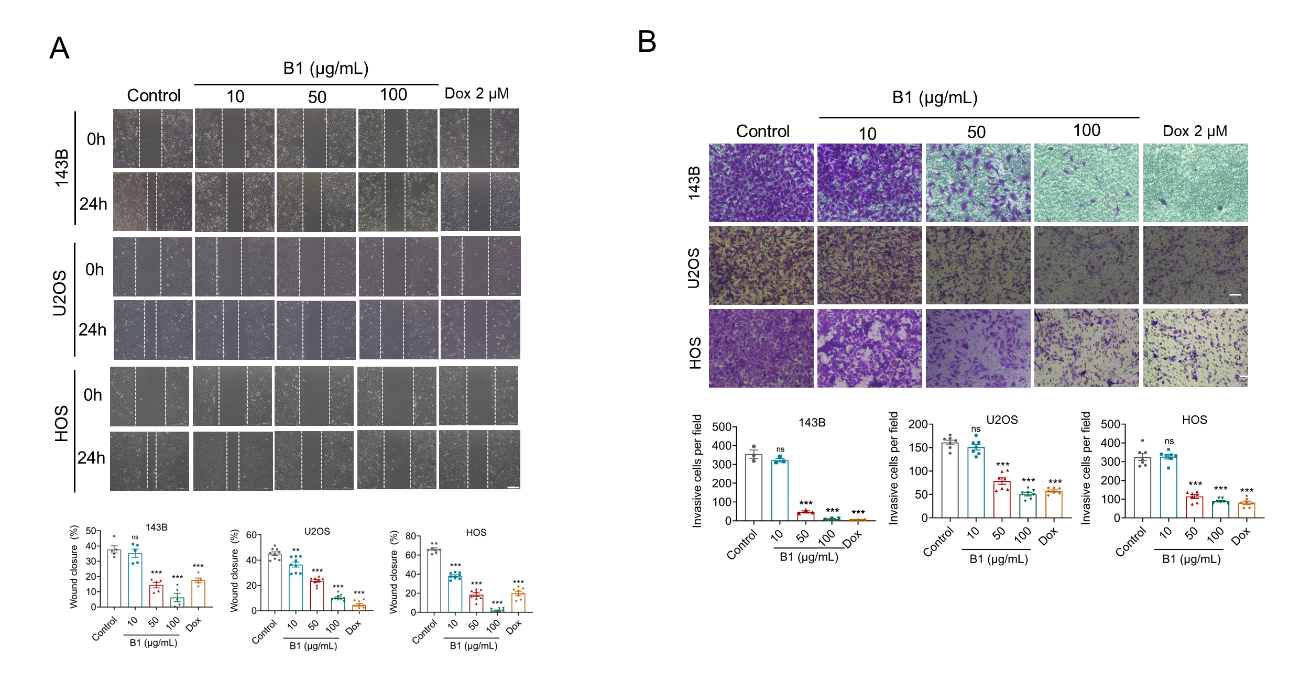
**

**Figure S1.** (A) Scratch test showing the migration ability of 143B (n=5), U2OS (n=9), and HOS (n=8) cell lines at 0h and 24h. (B) Transwell assay for the invasion of 143B (n=3), U2OS (n=7), and HOS (n=7) cell lines with different concentrations of B1 and Dox treatments. Scale bar: 50 µm. Data were expressed as mean ± SEM. ns: no significant difference; ***P* < 0.01; ****P* < 0.001.

**Figure S2**

**
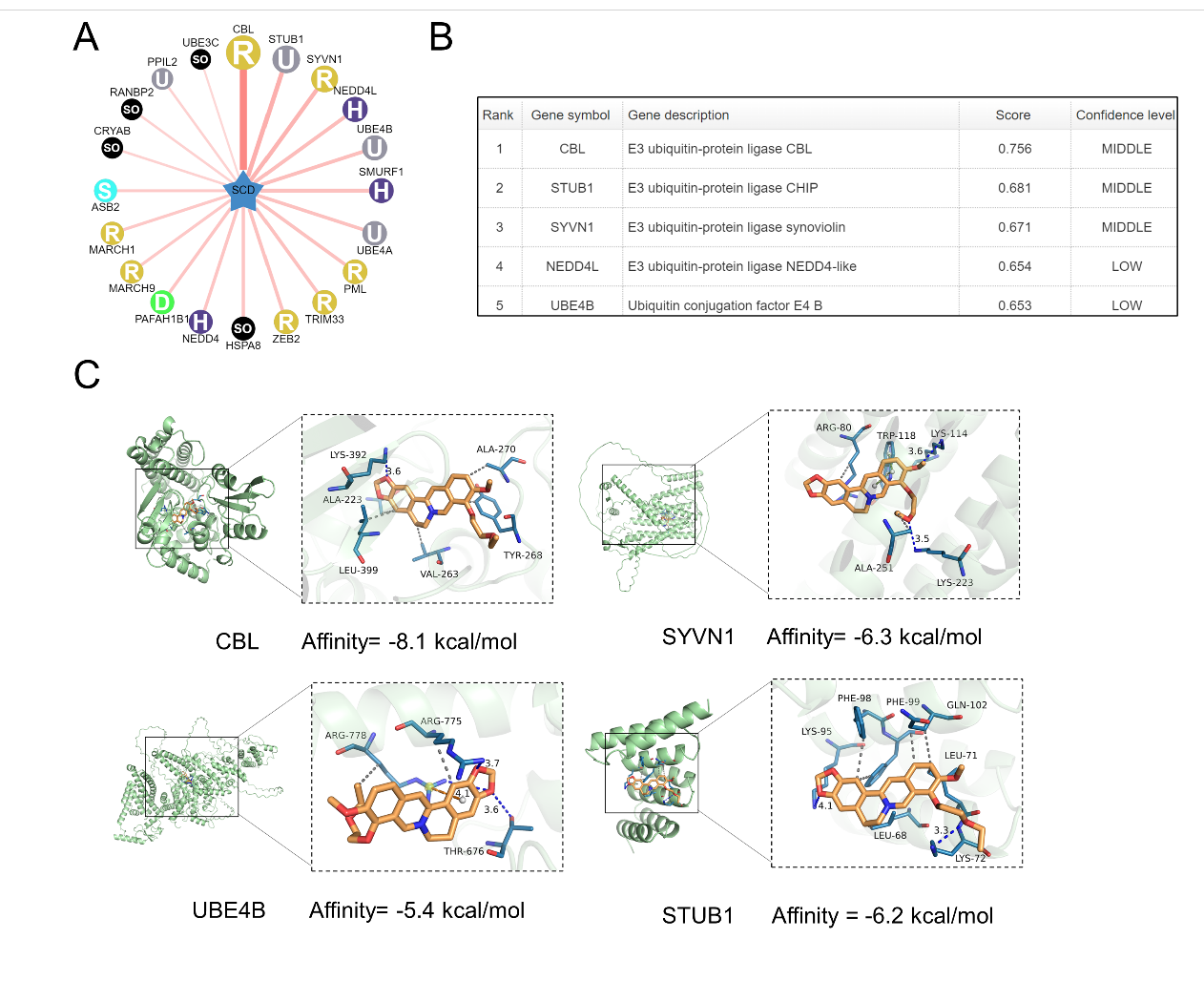
**

**Figure S2.** The table lists the top five ubiquitination ligase proteins in terms of score.

**Figure S3**


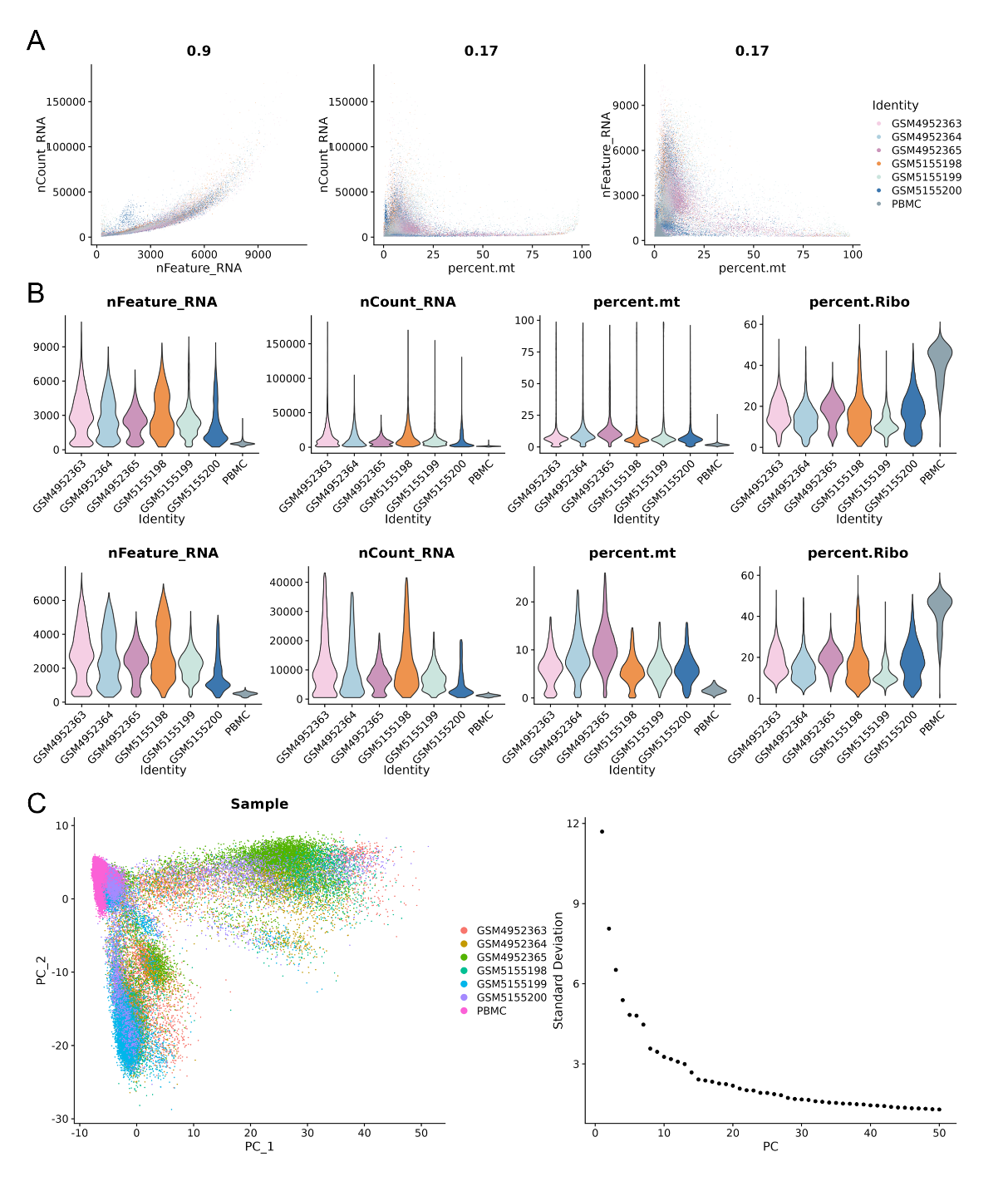


**Figure S3.** (A) Scatter plots of data quality control for different samples show the distribution of the number of detected genes in the samples. (B) Violin plots displaying the normalized expression levels of nFeature_RNA, nCount_RNA, percent.mt, and percent.Ribo for each sample as indicated, with two sets of plots showing different data representations. (C) t-SNE plot showing the distribution of different samples in each dataset, color-coded by sample type, along with a plot of standard deviation across principal components (PCs).

**Figure S4**


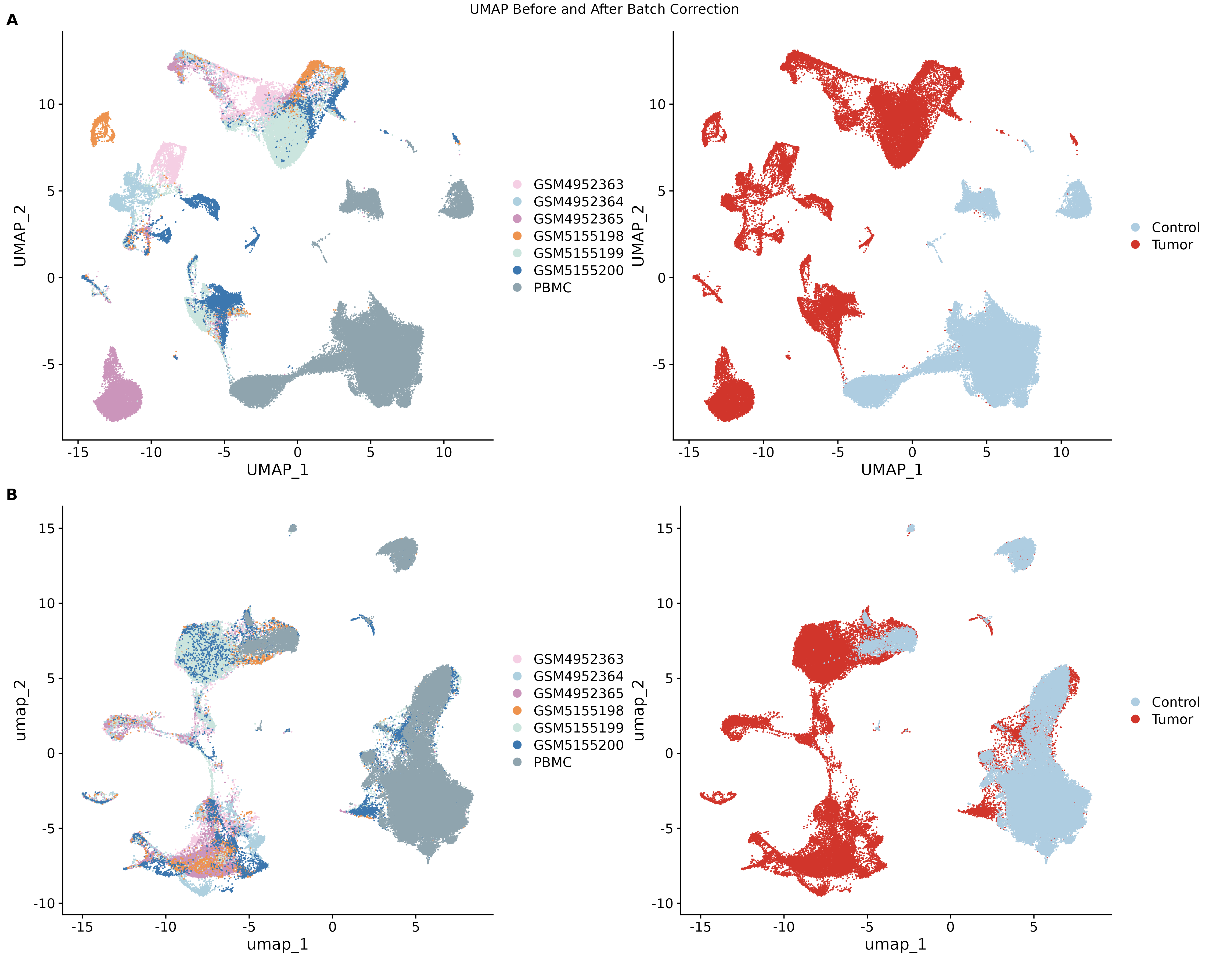


**Figure S4.** The UMAP plots show the distribution of cells in the UMAP space before and after batch correction as analyzed by Harmony, presenting the changes in the clustering of cells from different samples.

**Figure S5**

**
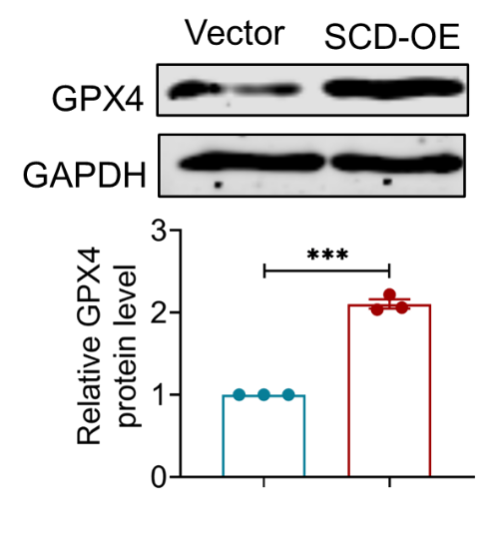
**

**Figure S5.** Western blot analysis of GPX4 protein in SCD overexpressed 143B cells (n=3). Data presented as mean ± SEM. *** *p* <0.001.

**Figure S6**

**
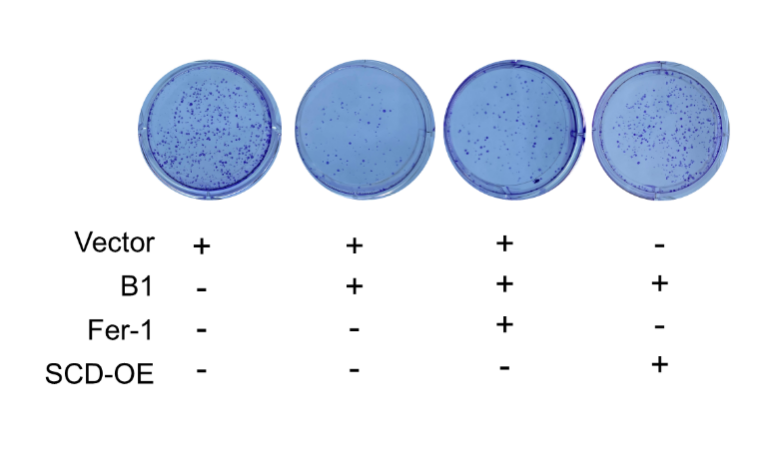
**

**Figure S6.** Representative images of the cloning wells of 143B cells (n=3).

**Figure S7**


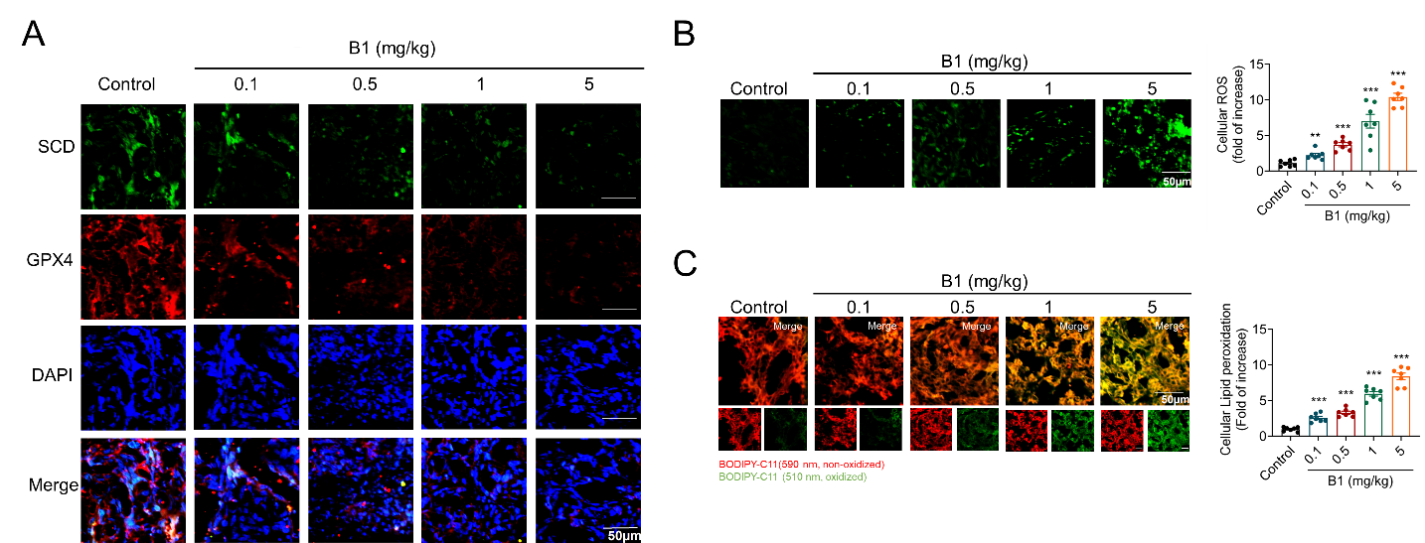


**Figure S7.** (A) Immunofluorescence of osteosarcoma tissue grown treated with 0, 0.1, 0.5, 1, 5 mg/ml B1 as indicated. SCD (green) and GPX4 (red) are indicated. n=7. (B) ROS detection of osteosarcoma tissue treated with 0, 0.1, 0.5, 1, 5 mg/ml B1 by fluorescence staining. n=7. (C) Confocal imaging of C11-BODIPY loaded osteosarcoma tissue treated with different concentrations of B1. Red, reduced form of C11-BODIPY; Green, oxidized form of C11-BODIPY. n=7. Scale bar: 50 µm. Data presented as mean ± SEM. ** *p* <0.01, *** *p* <0.001.

**Figure S8**

**
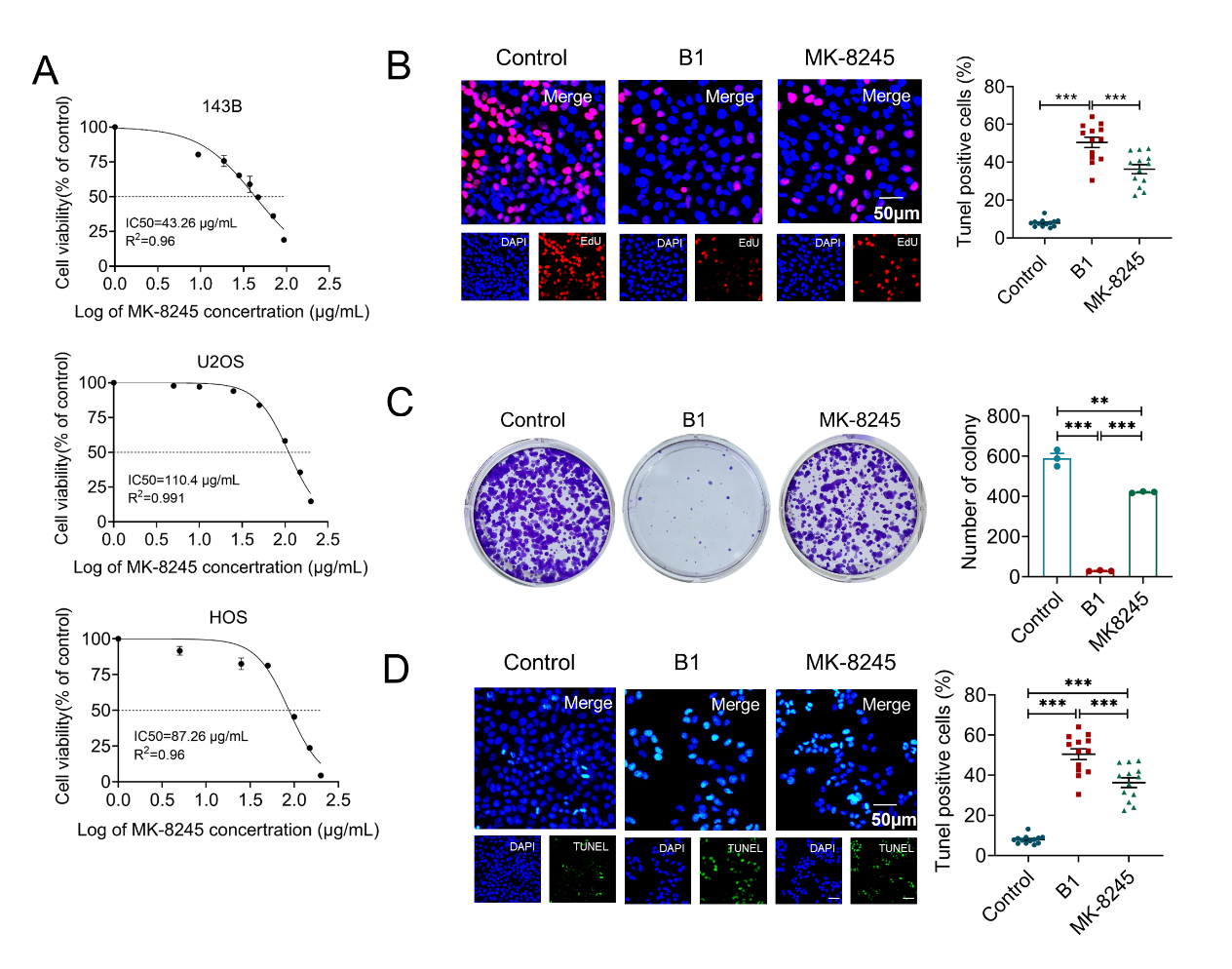
**

**Figure S8.** (A) Dose–response curves with IC50 values for MK-8245 (the inhibitor of SCD) in 143B, U2OS, and HOS cell lines. The results are expressed as a percentage of cell viability with no inhibitors. n=3. (B) Representative images of EdU staining of 143B treated with 50 µg/ml B1 or 50 µg/ml MK-8245. Cell nucleus exhibits blue and EdU labeling indicates replicating cells. n=12. (C) Effects of 50 µg/ml B1 or 50 µg/ml MK-8245 on cell proliferation in 143B with clone formation assay. n=3. (D) TUNEL staining renders the effect of 50 µg/ml B1 or 50 µg/ml MK-8245 on apoptosis in 143B. n=13. Data presented as mean ± SEM.*P < 0.05, *** p <0.001.

**Figure S9**

**
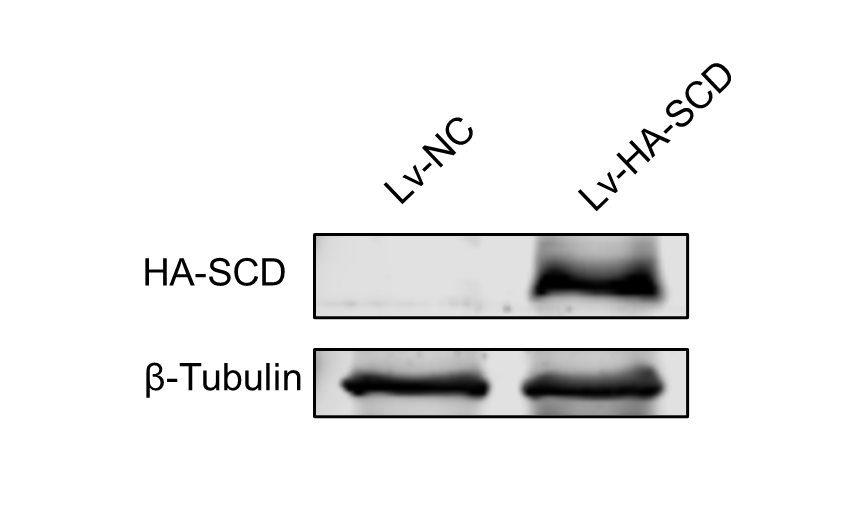
**

**Figure S9.** Transfection efficiency of SCD overexpression lentivirus was verified by Western blot.
